# p53 downregulates the Fanconi anaemia DNA repair pathway

**DOI:** 10.1038/ncomms11091

**Published:** 2016-04-01

**Authors:** Sara Jaber, Eléonore Toufektchan, Vincent Lejour, Boris Bardot, Franck Toledo

**Affiliations:** 1 Genetics of Tumour Suppression, Equipe Labellisée Ligue, Institut Curie, Centre de recherche, 26 rue d'Ulm, 75248 Paris Cedex 05, France; 2 Sorbonne Universités, UPMC Univ Paris 06, Paris, France; 3 CNRS UMR 3244, Paris, France; 4 PSL Research University, Paris, France

## Abstract

Germline mutations affecting telomere maintenance or DNA repair may, respectively, cause dyskeratosis congenita or Fanconi anaemia, two clinically related bone marrow failure syndromes. Mice expressing p53^Δ31^, a mutant p53 lacking the C terminus, model dyskeratosis congenita. Accordingly, the increased p53 activity in *p53*^*Δ31/Δ31*^ fibroblasts correlated with a decreased expression of 4 genes implicated in telomere syndromes. Here we show that these cells exhibit decreased mRNA levels for additional genes contributing to telomere metabolism, but also, surprisingly, for 12 genes mutated in Fanconi anaemia. Furthermore, *p53*^*Δ31/Δ31*^ fibroblasts exhibit a reduced capacity to repair DNA interstrand crosslinks, a typical feature of Fanconi anaemia cells. Importantly, the p53-dependent downregulation of *Fanc* genes is largely conserved in human cells. Defective DNA repair is known to activate p53, but our results indicate that, conversely, an increased p53 activity may attenuate the Fanconi anaemia DNA repair pathway, defining a positive regulatory feedback loop.

## Abstract (web summary)

P53 is regarded as the guardian of the genome, however it is known that mice with increased p53 activity display characteristics of dyskeratosis congenita. Here the authors show that increased p53 activity leads to the repression of telomere maintenance and DNA repair genes.

## Graphical abstract

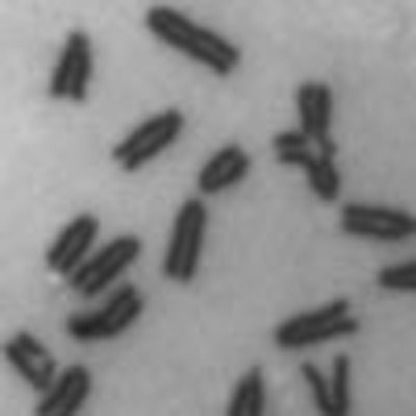

Inherited bone marrow failure syndromes are a set of clinically related yet heterogeneous disorders in which at least one haematopoietic cell lineage is significantly reduced. Among them, Fanconi anaemia (FA) and dyskeratosis congenita (DC) are caused by germline mutations in key cellular processes, that is, DNA repair and telomere maintenance, respectively^1^.

We recently found that *p53*^*Δ31/Δ31*^ mice, expressing a mutant p53 lacking its C-terminal domain, die rapidly after birth with a complete set of features of the telomere syndrome DC, including aplastic anaemia, pulmonary fibrosis, oral leukoplakia, skin hyperpigmentation, nail dystrophy and short telomeres^2^. Loss of the p53 C terminus increases p53 activity in mouse embryonic fibroblasts (MEFs) and in most tested tissues^2^,^3^, and *p53*^*Δ31/Δ31*^ MEFs exhibited decreased messenger RNA (mRNA) levels for 4 out of 10 genes implicated in telomere syndromes (*Dkc1*, *Rtel1*, *Tinf2* and *Terf1*). Nutlin, a drug that prevents the Mdm2 ubiquitin ligase from interacting with p53, allowed to confirm that p53 activation leads to the downregulation of these four genes. These data revealed that p53 plays a major role in telomere metabolism.

We previously focused on the potential p53-mediated regulation of genes mutated in DC (*Dkc1*, *Rtel1* and *Tinf2*) or implicated in aplastic anaemia, a milder form of telomere syndrome (*Terf1*)^4^. As a striking evidence for the clinical relevance of our mouse model, patients with severe DC who carry mutations affecting PARN, a negative regulator of p53, were recently shown to exhibit decreased DKC1, RTEL1 and TERF1 mRNA levels^5^. Importantly, however, tens of proteins are thought to be involved in the regulation of telomeres (reviewed in ref. 6). Thus, it remained possible that the impact of p53 on telomere-related genes was underestimated in our previous study. Here we tested whether p53 affects the expression of 42 additional genes implicated in telomere metabolism, and found 7 genes that are downregulated in *p53*^*Δ31/Δ31*^ cells. Importantly, some of these p53-regulated genes are involved in the FA DNA repair pathway. This was particularly intriguing because *Rtel1*, one of the four telomere-related genes we previously found regulated by p53, encodes a Fancj-like helicase^7^. These observations led us to evaluate whether p53 regulates more genes belonging to the FA pathway, and whether *p53*^*Δ31/Δ31*^ cells exhibit characteristic features of FA cells. We found that murine p53 downregulates 12 *Fanc* genes, that human p53 downregulates 9 *FANC* genes and that the capacity to repair DNA interstrand crosslinks is attenuated upon p53 activation. These data reveal an unexpected role for p53 in downregulating the FA DNA repair pathway, which may help to understand the pathological processes implicated in FA, and suggest therapeutic strategies against tumour cells that retain a functional p53 pathway.

## Results

### Expression of telomere-related genes in *p53* ^*Δ31/Δ31*^ cells

Our initial aim was to test whether, besides the four genes previously identified^2^, p53 could regulate other genes that might contribute to the telomere phenotype of *p53*^*Δ31/Δ31*^ mice. We therefore compared, in unstressed *p53*^*−/−*^, wild-type (WT) and *p53*^*Δ31/Δ31*^ fibroblasts, mRNA levels for 42 candidate genes reported to be relevant to telomere metabolism. Candidates included genes implicated in telomere syndromes (*Acd/Tpp1*, *Apollo/Snm1b*, *C16orf57/Mpn1/Usb1*, *Naf1*, *Obfc1/Stn1*, *Parn* and *Sbds*)^5^,^6^,^8^,^9^,^10^; genes mutated in diseases not primarily associated with telomere biology but for which telomere dysfunction or DC-like features were reported (*Dnmt3b*, *Fancd2* and *Recql4)*^6^; genes encoding proteins of complexes involved in telomere biology, that is, the telomerase (*Gar1/Nola1*, *Ruvbl1* and *Ruvbl2*), shelterin (*Pot1a* and *Pot1b*, *Rap1/Terf2ip*, and *Terf2*), CST (*Ten1*) and CIA (*Ciao1*, *Iop1/Narfl*, *Mip18* and *Mms19*) complexes, as well as Cajal bodies (*Coilin* and *Hot1*)^6^,^11^, or proteins otherwise proposed to participate in telomere replication or maintenance (*Artemis/Snm1c*, *Blm*, *Csb/Ercc6*, *Dek*, *Dna2*, *Ercc3/Xpb*, *Ercc4/Fancq/Xpf*, *Fancc, Fen1*, *Lmna/Progerin, Nbs1, Pim1*, *Slx4/Fancp*, *Timeless*, *Tnks1*, *Tnks1bp1*, *Upf1* and *Wrn*)^6^,^12^,^13^,^14^,^15^,^16^,^17^,^18^,^19^,^20^,^21^,^22^,^23^,^24^,^25^,^26^.

**Figure 1:**
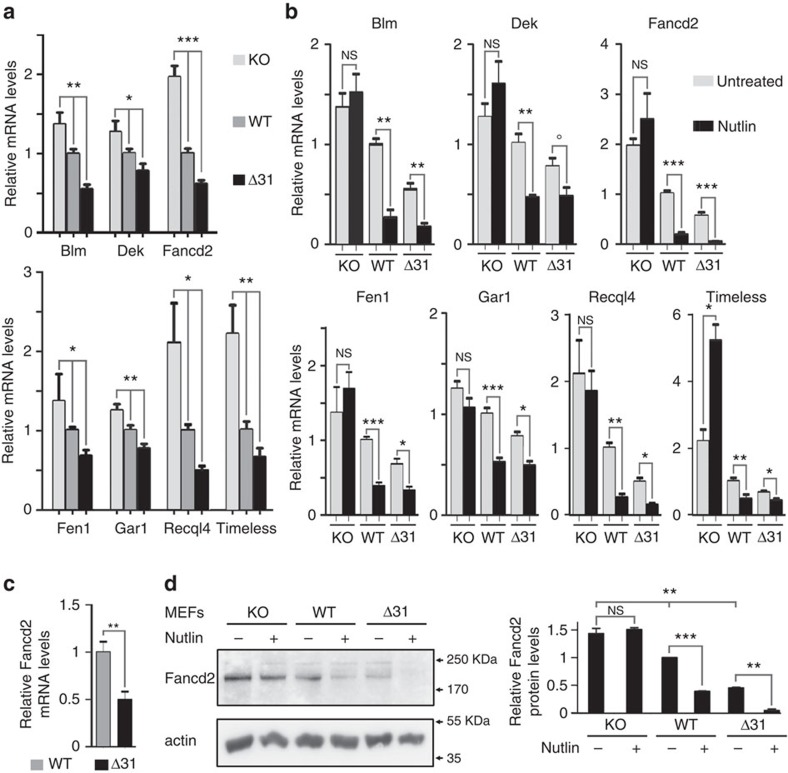
p53 activation leads to the downregulation of seven telomere-related genes. (**a**) A comparison of *p53*^*−/−*^, wild-type and *p53*^*Δ31/Δ31*^ cells suggests the p53-dependent regulation of *Blm, Dek, Fancd2, Fen1, Gar1, Recql4* and *Timeless*. RNAs, prepared from unstressed *p53*^*−/−*^ (KO), wild-type (WT) and *p53*^*Δ31/Δ31*^ (Δ31) MEFs, were used to compare the expression of 42 genes with a proposed impact on telomere metabolism. mRNAs were quantified using real-time PCR, normalized to control mRNAs, then the amount in WT cells was assigned a value of 1. Shown here are the seven genes for which the mean mRNA levels were intermediate in WT cells compared with *p53*^*−/−*^ and *p53*^*Δ31/Δ31*^ cells, with significant differences between the means according to one-way analysis of variance (ANOVA). For the 35 genes that did not match these criteria, see Supplementary Fig. 1. Results from ⩾3 independent experiments. (**b**) The seven genes are downregulated upon p53 activation. mRNAs were quantified in *p53*^*−/−*^, WT and *p53*^*Δ31/Δ31*^ MEFs, untreated or treated with 10 μM Nutlin for 24 h. Results from ⩾3 independent experiments. °*P*=0.059. (**c**) Fancd2 mRNAs are decreased in the bone marrow cells (BMCs) of *p53*^*Δ31/Δ31*^ mice. Fancd2 mRNAs were quantified from the BMCs of nine WT and six *p53*^*Δ31/Δ31*^ mice. (**d**) p53 activation leads to decreased Fancd2 protein levels. Protein extracts, prepared from untreated or Nutlin-treated MEFs, were immunoblotted with antibodies against Fancd2 and actin. On the left, a typical western blot is shown; on the right, bands from two western blots were quantified and the amount of Fancd2 in unstressed WT cells was assigned a value of 1. In all figures, means+s.e.m. are shown; ***P≤0.001, **P≤0.01, *P≤0.05, NS, not significant by analysis of variance or Student's *t*-tests.

For a gene to be a good candidate, we considered that the mean (from three to four independent experiments) of its mRNA levels in unstressed WT cells should fall between the means measured in *p53*^*−/−*^ and *p53*^*Δ31/Δ31*^ cells; and that the means for the three genotypes should be statistically different according to an analysis of variance. Out of the 42 genes, 7 fulfilled these criteria: *Blm*, *Dek*, *Fancd2*, *Fen1*, *Gar1*, *Recql4* and *Timeless* (Fig. 1a; Supplementary Fig. 1). Because *RECQL4* was shown to be downregulated by p53 in human cells^27^, the lower Recql4 mRNAs in *p53*^*Δ31/Δ31*^ cells were not surprising. The decreased mRNA levels for the six other genes were not anticipated however. To specifically assay for a p53-dependent regulation, we next compared the effects of Nutlin, a drug that activates p53 by preventing its interaction with the ubiquitin ligase Mdm2. Results clearly indicated that p53 activation leads to the downregulation of these genes (Fig. 1b).

Importantly, the finding that p53 downregulates *Gar1*, which encodes a component of the telomerase complex, strengthened our previous conclusion that p53 plays a significant role in telomere biology. However, *Fancd2* appeared as the gene whose expression was most markedly affected by p53 activation (Fig. 1b). This was surprising because, even if primary cells from patients with a *FANCD2* mutation may exhibit telomere dysfunction^28^, these patients are diagnosed with FA, a syndrome primarily characterized by defects in DNA repair. This led us to further analyse the p53-dependent regulation of *Fancd2*. We first verified that the relative decrease in Fancd2 mRNA levels were observed *in vivo*, in bone marrow cells (BMCs) from *p53*^*Δ31/Δ31*^ mice (Fig. 1c). We next tested whether the p53-dependent regulation of *Fancd2* detected by quantitative PCR had an impact on Fancd2 protein levels. Lower Fancd2 protein levels were observed in unstressed *p53*^*Δ31/Δ*31^ cells compared with unstressed *p53*^*−/−*^ or WT cells, and Nutlin treatment led to a decrease in Fancd2 proteins only in WT and *p53*^*Δ31/Δ31*^ MEFs, in complete agreement with quantitative PCR data (Fig. 1d; Supplementary Fig. 2).

### p53 activation leads to increased E2F4 binding at *Fancd2*

**Figure 2:**
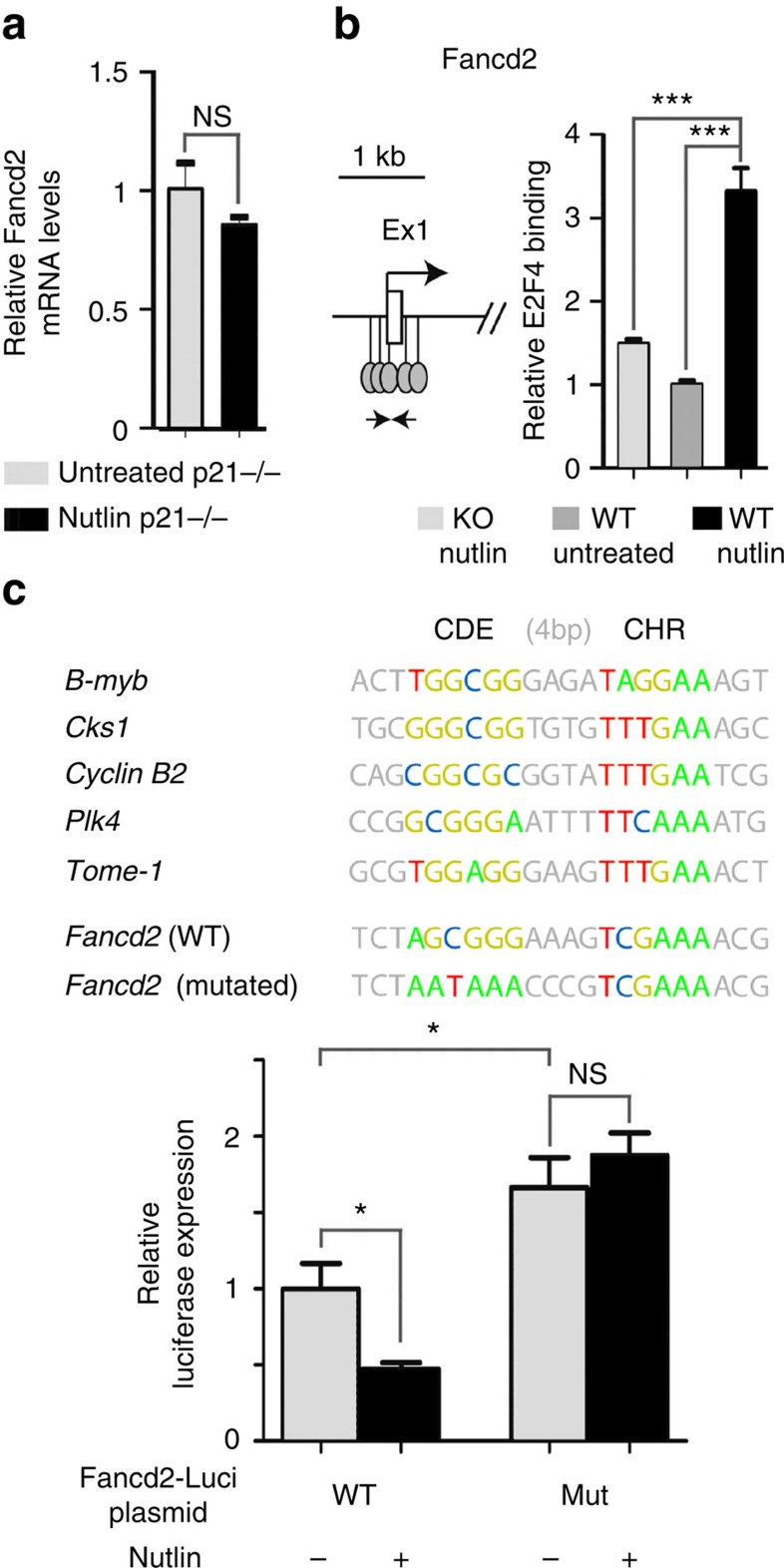
p53 activation promotes the binding of transcriptional repressor E2F4 at the *Fancd2* gene. (**a**) p21 is required for the downregulation of *Fancd2*. mRNAs from untreated or Nutlin-treated *p21*^*−/−*^ MEFs were quantified. Results from three independent experiments. (**b**) Increased E2F4 binding at the *Fancd2* promoter upon p53 activation. A map surrounding the *Fancd2* transcription start site (TSS) is shown on the left (white box: UTR (Ex1: exon 1); lollipops: putative E2F4-binding sites according to ref. 70 (Supplementary Fig. 3); arrows: ChIP PCR primers), and ChIP data on the right. ChIP assay for E2F4 binding was performed in Nutlin-treated *p53*^*−/−*^ MEFs, and untreated or Nutlin-treated WT MEFs, with an antibody against E2F4 or rabbit IgG as a negative control. Immunoprecipitates were quantified using real-time PCR, fold enrichment was normalized to data over an irrelevant region, then E2F4 binding at *Fancd2* in untreated WT cells was given a value of 1. Data from two independent ChIP experiments, each quantified in triplicates. (**c**) The p53-dependent regulation of *Fancd2* occurs via a CDE/CHR motif. CDE/CHR motifs are required for gene repression by an E2F4-containing DREAM complex^32^. These motifs consist of a 6-bp long GC-rich CDE site (bound by E2F4) located 4-bp upstream of a 6-bp long AT-rich CHR site. On top, CDE/CHR motifs regulating the expression of five mouse genes are presented, as well as a putative CDE/CHR motif 23–38-bp downstream of the mouse *Fancd2* TSS, and its mutated counterpart (with mutations in the CDE). Below, a 2-kb fragment centred around the *Fancd2* TSS, containing a WT or mutant CDE/CHR, was cloned upstream a Luciferase gene and transfected into NIH-3T3 cells, treated or not with Nutlin, then Luciferase activity was measured after 24 h. Although the cell cycle kinetics of cells transfected with either plasmid were identical (Supplementary Fig. 4), Nutlin led to decreased luciferase activity only with the construct containing a WT CDE/CHR motif. Mutation of the putative CDE site increased Luciferase basal expression, and abrogated the effect of Nutlin. Results from three independent experiments. In all figures, means+s.e.m. are shown; ***P≤0.001, *P≤0.05, NS, not significant by Student's *t*-test.

The p53-mediated downregulation of many genes requires the cdk inhibitor p21, and occurs through the recruitment, upon p53 activation, of E2F4 repressive complexes at their promoters^29^,^30^. Notably, this mechanism would account for the p53-dependent regulation of cell cycle genes whose promoters contain CDE/CHR regulatory motifs^31^,^32^,^33^. Consistent with this mechanism, p53 activation had no effect on Fancd2 mRNA levels in *p21*^*−/−*^ cells (Fig. 2a), and chromatin immunoprecipitation (ChIP) experiments with an antibody against E2F4 indicated increased E2F4 binding at the *Fancd2* promoter in Nutlin-treated WT cells, compared with unstressed WT or Nutlin-treated *p53*^*−/−*^ cells (Fig. 2b; Supplementary Fig. 3). Of note, ChIP assays for E2F4 binding at the *Fancd2* promoter could not be performed in *p53*^*Δ31/Δ31*^ MEFs because their accelerated senescence^2^ prevented the recovery of sufficient amounts of chromatin, but it is likely that the p53/p21/E2F4 pathway operates similarly in *p53*^*Δ31/Δ31*^ cells. We next identified a candidate CDE/CHR motif in the *Fancd2* promoter, and mutation of the CDE element (typically bound by E2F4) abolished the Nutlin-dependent repression of this promoter in NIH-3T3 cells (Fig. 2c), independently of cell cycle dynamics (Supplementary Fig. 4). Thus, although the expression of *Fancd2* is known to vary during the cell cycle^34^, the differences in Fancd2 mRNA levels observed between WT and *p53*^*Δ31/Δ31*^ MEFs would not simply result from differences in G1/S ratios^2^. Rather, our results indicate that p53 activation promotes the recruitment of E2F4 at the *Fancd2* gene, and that E2F4 plays a major role in the repression of *Fancd2*.

In the experiments above, p53 activation resulted from a treatment with Nutlin, a molecule that acts as a specific Mdm2 inhibitor. We next tested whether similar results could be obtained in response to DNA damage, by evaluating the effects of doxorubicin, a clastogenic anticancer agent. Doxorubicin treatment led to decreased Fancd2 mRNA and protein levels in WT and *p53*^*Δ31/Δ31*^ cells, but not *p53*^*−/−*^ MEFs (Supplementary Fig. 5a,b). Furthermore, we observed increased E2F4 binding at the *Fancd2* promoter in doxorubicin-treated WT cells, compared with unstressed WT or doxorubicin-treated *p53*^*−/−*^ cells (Supplementary Fig. 5c). Thus, both Nutlin and doxorubicin lead to p53 activation and consecutive *Fancd2* downregulation.

Interestingly, the *Blm* and *Fen1* genes, also downregulated by p53 (Fig. 1b), respectively, encode an helicase that associates with Fanc proteins in a multienzyme complex^35^, and an endonuclease stimulated by a Fanc protein^36^. Furthermore, *Rtel1*, one of the four telomere-related genes we previously found regulated by p53 (ref. 2), encodes a Fancj-like helicase^7^. This led us to further evaluate the impact of p53 activation on the FA DNA repair pathway.

### p53 downregulates many *Fanc* genes

**Figure 3:**
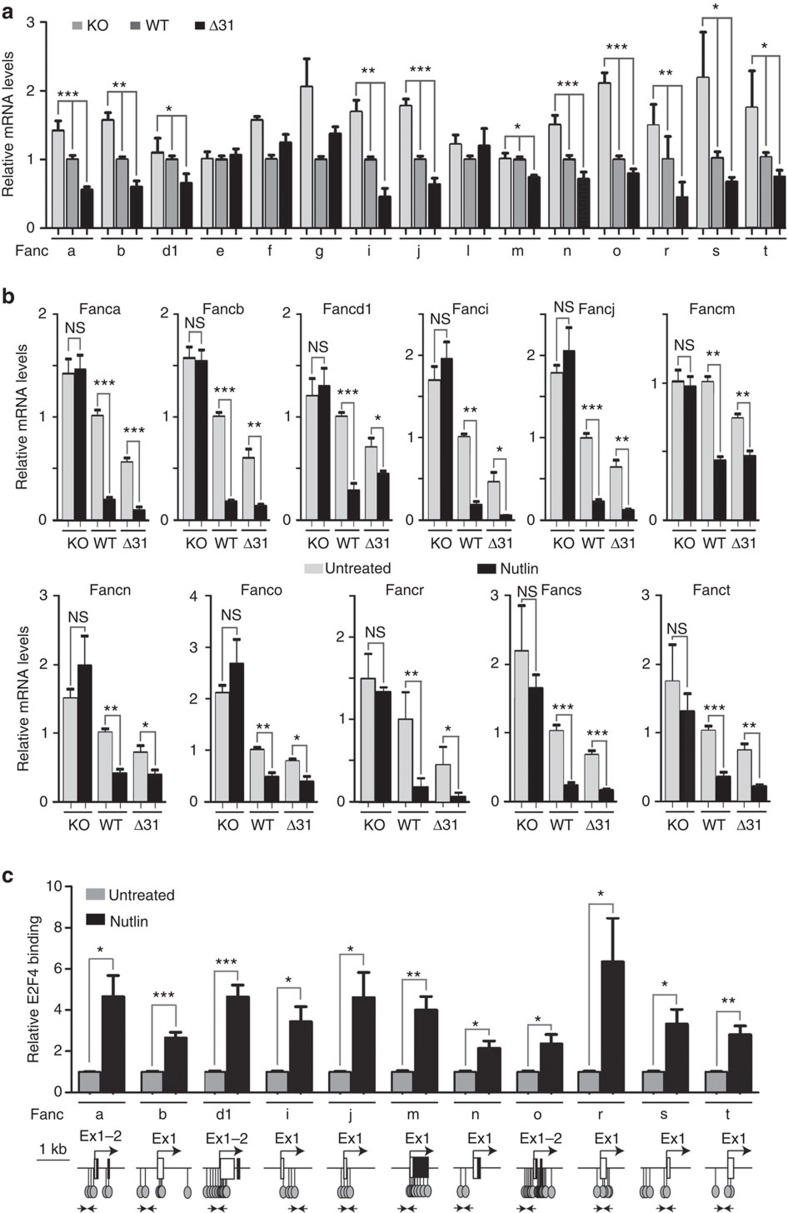
Several genes of the FA DNA repair pathway are downregulated upon p53 activation. (**a**) A comparison of *p53*^*−/−*^, wild-type and *p53*^*Δ31/Δ31*^ cells suggests a potential p53-dependent regulation for 11 additional genes of the Fanconi anaemia (FA) DNA repair pathway. mRNAs for the indicated *Fanc* genes were quantified as described in Fig. 1a, in four independent experiments. For 11 of the tested genes, mean mRNA levels were intermediate in WT cells compared with *p53*^*−/−*^ and *p53*^*Δ31/Δ31*^ cells, with statistical significance by one-way analysis of variance (ANOVA). (**b**) The 11 genes are downregulated on murine p53 activation. mRNAs for the indicated *Fanc* genes were quantified in untreated or Nutlin-treated MEFs. Results from three independent experiments. (**c**) Increased E2F4 binding at several *Fanc* promoters upon p53 activation. ChIP assay for E2F4 binding was performed in untreated or Nutlin-treated WT MEFs, with an antibody against E2F4 or rabbit IgG as a negative control. Immunoprecipitates were quantified using real-time PCR, fold enrichment was normalized to data over an irrelevant region, and then E2F4 binding in untreated WT cells was given a value of 1. Data are from two to three independent ChIP experiments, each quantified in triplicates. Below the ChIP data are represented, as in Fig. 2b, sequences around the TSS for each gene, putative E2F4-binding sites (lollipops), and primers used for ChIP assays (arrows). In all figures, means+s.e.m. are shown; ***P≤0.001, **P≤0.01, *P≤0.05, NS, not significant by ANOVA or Student's *t*-tests.

**Figure 4:**
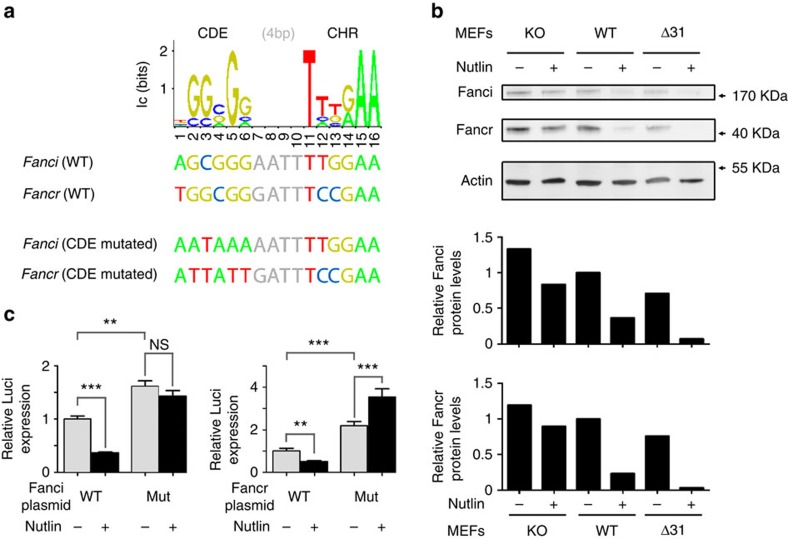
CDE/CHR motifs are important for the p53-dependent repression of *Fanci* and *Fancr*. (**a**) Identification of candidate CDE/CHR motifs in *Fanci* and *Fancr* with a positional frequency matrix. The CDE/CHR motifs in six mouse genes were used to define the positional frequency matrix shown on top, which was then used to identify candidate CDE/CHR motifs in *Fanci* and *Fancr* (for details, see Supplementary Fig. 7). The candidate CDE/CHRs map 38 (*Fanci*)- and 15 (*Fancr*)-bp downstream of the transcription start site (TSS) of each gene. Also shown here are the mutated CDE/CHRs that were tested in luciferase assays in **c**. (**b**) p53 activation leads to decreased Fanci and Fancr protein levels. Protein extracts, prepared from untreated or Nutlin-treated MEFs, were immunoblotted with antibodies against Fanci, Fancr and actin, then bands were quantified and the amounts of Fanci or Fancr proteins in unstressed WT cells were assigned a value of 1. (**c**) The p53-dependent regulation of *Fanci* and *Fancr* occurs via a CDE/CHR motif. For each gene, a 1-kb fragment centred around the TSS, containing a WT or mutant CDE/CHR, was cloned upstream of a luciferase gene and transfected into NIH-3T3 cells, treated or not with Nutlin, then luciferase activity was measured after 24 h. Mutation of the putative CDE site increased luciferase basal expression and abrogated the effect of Nutlin. Results from two independent experiments; means+s.e.m. are shown; ***P≤0.001, **P≤0.01, NS, not significant by Student's *t*-test.

Because the expression levels of four FA genes had been tested in our previous experiments—*Fancc*, *Fancd2*, *Fancp/Slx4* and *Fancq/Ercc4* (Fig. 1; Supplementary Fig. 1), we next compared, in unstressed *p53*^*−/−*^, WT and *p53*^*Δ31/Δ31*^ cells, mRNA levels for the 15 remaining FA genes. Strikingly, 11 were less expressed in *p53*^*Δ31/Δ31*^ cells (Fig. 3a). Again, Nutlin was used to confirm the p53-mediated downregulation of these genes (Fig. 3b). As for *Fancd2*, this p53-mediated downregulation required p21 (Supplementary Fig. 6), and p53 activation correlated with an increased binding of E2F4 near the transcription start site of each of these *Fanc* genes (Fig. 3c). We next used the sequence of six functional CDE/CHRs to define a positional frequency matrix, which was then used to search *in silico* for candidate CDE/CHRs near the E2F4-binding sites identified in ChIP assays. Using this approach, candidate CDE/CHR motifs were identified for 9 out of the 11 tested *Fanc* genes, with the best candidate motifs for *Fanci* and *Fancr* (Fig. 4a; Supplementary Fig. 7). These data led us to further analyse the p53-mediated regulation of *Fanci* and *Fancr*. We first verified that the relative decreases in Fanci and Fancr mRNA levels were observed *in vivo*, in BMCs from *p53*^*Δ31/Δ31*^ mice (Supplementary Fig. 8). We then found that p53 activation leads to decreased Fanci and Fancr protein levels *ex vivo* (Fig. 4b; Supplementary Fig. 9). Luciferase assays next showed that mutating the CDE site in each candidate CDE/CHR abolished the Nutlin-dependent repression of the *Fanci* and *Fancr* promoters (Fig. 4a,c).

We also observed that a 24-h long treatment with doxorubicin led to decreased Fanci and Fancr mRNA, and protein levels in WT and *p53*^*Δ31/Δ31*^ cells, but not *p53*^*−/−*^ MEFs (Supplementary Fig. 10a). Furthermore, the nine other *Fanc* genes downregulated by p53 on Nutlin treatment were also downregulated in a p53-dependent manner on treatment with doxorubicin (Supplementary Fig. 10b). We then searched for confirmation of our results by analysing the data recently reported by Younger *et al*., who performed a genomic analysis that integrated transcriptome-wide expression levels, genome-wide p53-binding profiles and chromatin state maps to characterize the regulatory role of p53 in response to DNA damage^37^. Although this approach was designed to identify direct p53 targets, we reasoned that genes regulated by p53 indirectly, via p21/E2F4, might also be detected in their transcriptome-wide expression data. These experiments were performed on *p53*^*−/−*^ and WT MEFs, treated or not with doxorubicin for 6 h (ref. 37), and our previous time-course experiments with Nutlin suggested that 6 h might be sufficient to observe a partial p53-mediated trancriptional downregulation^2^. Thus, we extracted the data of Younger *et al*. (Gene Expression Omnibus # GSE55727) to analyse the expression of the 12 *Fanc* genes that we had found downregulated by p53. In agreement with our results, this analysis showed that doxorubicin led to an overall decrease in the expression of *Fanc* genes in WT, but not *p53*^*−/−*^ MEFs (Supplementary Fig. 11).

Transcriptome data mining was also used to find whether the downregulation of *Fanc* genes could correlate with p53 activation in haematopoietic cells. The Homeobox (Hox) transcription factors are important regulators of normal and malignant haematopoiesis, because they control proliferation, differentiation and self-renewal of haematopoietic cells. We analysed the data of Muntean *et al*. (Gene Expression Omnibus # GSE21299), who immortalized murine BMCs by transduction with Hoxa9-ER cells in the presence of tamoxifen (4-OHT), and observed that they undergo myeloid differentiation 5 days after 4-OHT withdrawal^38^. We found this differentiation to correlate with an induction of genes known to be transactivated by p53 (*Cdkn1A/p21*, *Mdm2* and *Fas*), and with the downregulation of *Fanc* genes (Supplementary Fig. 12).

In sum, we found that 12 genes of the FA DNA repair pathway are downregulated by p53 via a p21/E2F4 pathway, and identified CDE/CHR motifs that are crucial for this regulation for three of these genes. Importantly, the genes are downregulated by p53 in response to Mdm2 inhibition or DNA damage, or on haematopoietic cell differentiation, and encode proteins involved in all parts of the FA DNA repair pathway, that is, proteins that belong to the FA core complex (*Fanca*, *Fancb* and *Fancm*) and its accessory protein (*Fanct/Ube2t*), the pivotal ID2 complex (*Fancd2* and *Fanci*), or downstream effector proteins (*Fancd1/Brca2*, *Fancj/Bach1/Brip1*, *Fancn/Palb2*, *Fanco/Rad51c, Fancr/Rad51* and *Fancs/Brca1*)^39^,^40^,^41^,^42^. Together, these data suggested an important role for p53 in regulating the FA pathway.

### p53 activation attenuates the repair of specific DNA lesions

**Figure 5:**
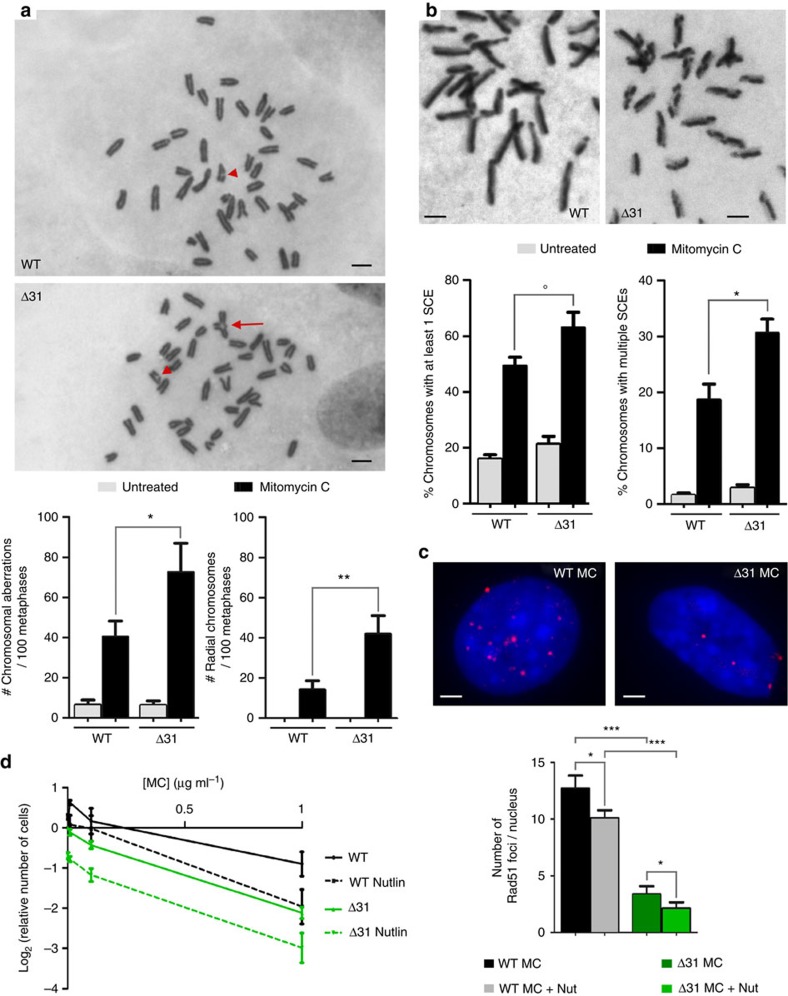
A decreased capacity to repair mitomycin C-induced DNA lesions in cells with increased p53 activity. (**a**) *p53*^*Δ31/Δ31*^ MEFs exhibit increased frequencies of mitomycin C-induced chromosomal aberrations. Frequencies of total chromosomal aberrations, or tri- and quadri-radial chromosomes, were determined in wild-type (WT) and *p53*^*Δ31/Δ31*^ (Δ31) MEFs at passage 3, untreated or after treatment with mitomycin C (MC). On top, typical examples of MC-treated WT and Δ31 metaphases presenting chromosomal aberrations (arrowheads: chromosome breaks; arrow: radial chromosome; scale bars, 2 μm). Below, results were plotted from 107 (WT untreated), 99 (WT MC-treated), 112 (Δ31 untreated) and 98 (Δ31 MC-treated) metaphases. To prevent any potential bias, cell preparations were dropped onto code-labelled slides (to mask the genotypes of cells to be analysed) and the same metaphases were independently observed by two experimenters. (**b**) *p53*^*Δ31/Δ31*^ MEFs exhibit increased frequencies of MC-induced sister chromatid exchanges. As in **a**, an unbiased procedure was used to determine the percentage of chromosomes presenting one or several sister chromatid exchanges (SCEs). On top, representative examples of chromosomes from MC-treated WT (left) or *p53*^*Δ31/Δ31*^ (right) metaphases displaying SCEs (scale bars, 2 μm). Below, results plotted from an analysis of 3,013 (WT untreated), 1,287 (WT MC treated), 1,905 (Δ31 untreated) and 340 (Δ31 MC treated) chromosomes. °*P*=0.059. (**c**) p53 activation correlates with a decreased capacity to form Rad51 foci in response to mitomycin C. Rad51 foci were counted in cells treated with MC or MC+Nutlin. On top, typical nuclei are shown (scale bars, 2 μm); below, results from >300 nuclei per genotype. The reduced capacity to form Rad51 foci might result from p53-dependent decreases in the expression of *Fancr/Rad51* as well as other *Fanc* genes. (**d**) Effects of p53 activation on the cellular sensitivity to MC. Cells were treated or not with Nutlin 2.5 μM for 24 h, then with MC at 0, 0.01, 0.1 and 1 μg ml^−1^ for 48 h, then counted. For each genotype, the final number of untreated cells was given a value of 1 and used as reference. Results from three experiments. Means+s.e.m. are shown; ***P≤0.001, **P≤0.01, *P≤0.05 by Student's *t*-test.

**Figure 6:**
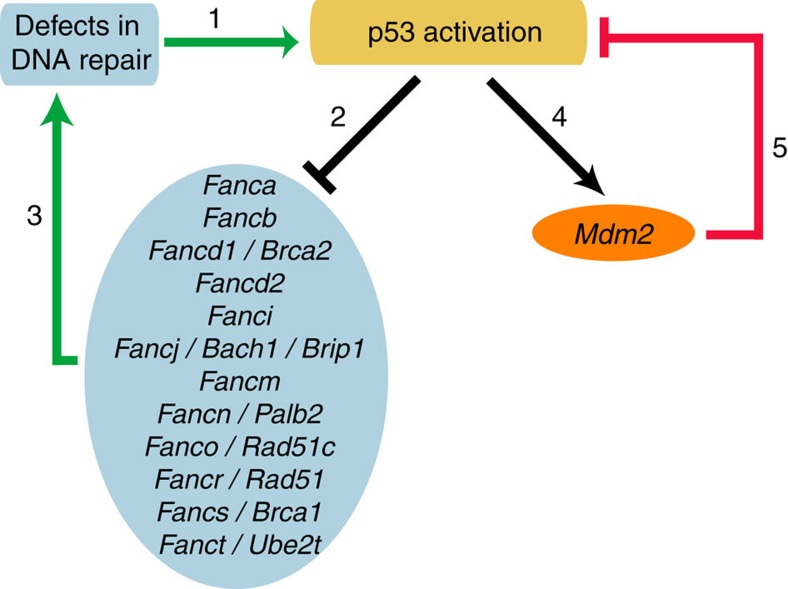
A simplified model of p53 regulation by a bipolar feedback system may account for the attenuated DNA repair capacity of *p53*^*Δ31/Δ31*^ MEFs. Defects in DNA repair activate p53 (1), and activated p53 downregulates several FA genes (2), which would attenuate the FA pathway and cause partial defects in DNA repair (3), hence defining a positive-feedback loop (in green). In unstressed WT MEFs, this positive-feedback loop would be efficiently counterbalanced by the negative-feedback loop (in red) between p53 and its major inhibitor, the ubiquitin ligase Mdm2 (4 and 5). In *p53*^*Δ31/Δ31*^ MEFs however, the p53^Δ31^ protein is more abundant, indicating that its interaction with Mdm2 is decreased^2^. Hence, the p53/Mdm2 negative-feedback loop is enfeebled in *p53*^*Δ31/Δ31*^ MEFs (or in *Mdm2*^*+/*−^ *Mdm4*^*+/**Δ**E6*^ MEFs), which would lead to a stronger p53/FA positive-feedback loop and thus to a reduced capacity to repair mitomycin C-induced DNA lesions. In both WT and *p53*^*Δ31/Δ31*^ cells, Nutlin specifically affects the p53/Mdm2 negative-feedback loop, which would further increase the cellular sensitivity to mitomycin C.

A typical feature of FA cells is their inability to repair DNA interstrand crosslinks, as evidenced by an increased frequency of chromosomal aberrations, and more specifically tri- and quadri-radial chromosomes, after exposure to mitomycin C (MC)^39^. We compared the effects, on WT and *p53*^*Δ31/Δ31*^ cells, of a 48-h treatment with 50 nM MC. Such a treatment procedure was previously reported to differentially affect WT MEFs and MEFs with an impaired FA pathway^43^. Interestingly, we found that this procedure led to a rather subtle induction of p53 (suggested by a limited increase in p21 transactivation), which correlated with a twofold decrease in Fancd2 mRNA expression in *p53*^*Δ31/Δ31*^ MEFs, but no significant alteration of Fancd2 mRNA levels in WT cells (Supplementary Fig. 13). We next determined the frequencies of all types of chromosomal aberrations, or of radial chromosomes, in WT and *p53*^*Δ31/Δ31*^ cells before or after treatment with MC. In untreated cells, no significant difference was found between the two genotypes. Strikingly, however, chromosomal aberrations, and particularly radial chromosomes, were more frequent in *p53*^*Δ31/Δ31*^ cells after treatment with MC, consistent with a decreased capacity to repair interstrand crosslinks in the mutant cells (Fig. 5a). Accordingly, chromosomes with sister chromatid exchanges were also more frequent in MC-treated *p53*^*Δ31/Δ31*^ cells than in WT cells (Fig. 5b). These results suggested that the FA DNA repair pathway is attenuated in *p53*^*Δ31/Δ31*^ cells, presumably because these cells exhibit an increased p53 activity. Consistent with this, *p53*^*Δ31/Δ31*^ cells exhibited a decreased capacity to form Rad51 foci and an increased sensitivity to MC, and the pretreatment of cells with Nutlin appeared to further impact on these cellular phenotypes (Fig. 5c,d). Further evidence that the decreased DNA repair in *p53*^*Δ31/Δ31*^ cells resulted from increased p53 activity (rather than a loss of the p53 CTD *per se*) came from analysing *Mdm2*^*+/*−^ *Mdm4*^*+/**Δ**E6*^ MEFs. These MEFs express a WT p53 protein, but exhibit an increased p53 activity due to lower levels of p53 inhibitors^44^,^45^. Like *p53*^*Δ31/Δ31*^ MEFs, *Mdm2*^*+/*−^ *Mdm4*^*+/**Δ**E6*^ cells were more sensitive than WT cells to MC (Supplementary Fig. 14). In sum, a defective FA DNA repair pathway is known to activate p53 (ref. 46), but these results indicate that an increased p53 activity might reduce the expression of several FA genes and attenuate the FA DNA repair pathway. Taken together, these data indicate the existence of a positive regulatory feedback loop (Fig. 6).

### Human p53 also regulates FA genes

**Figure 7:**
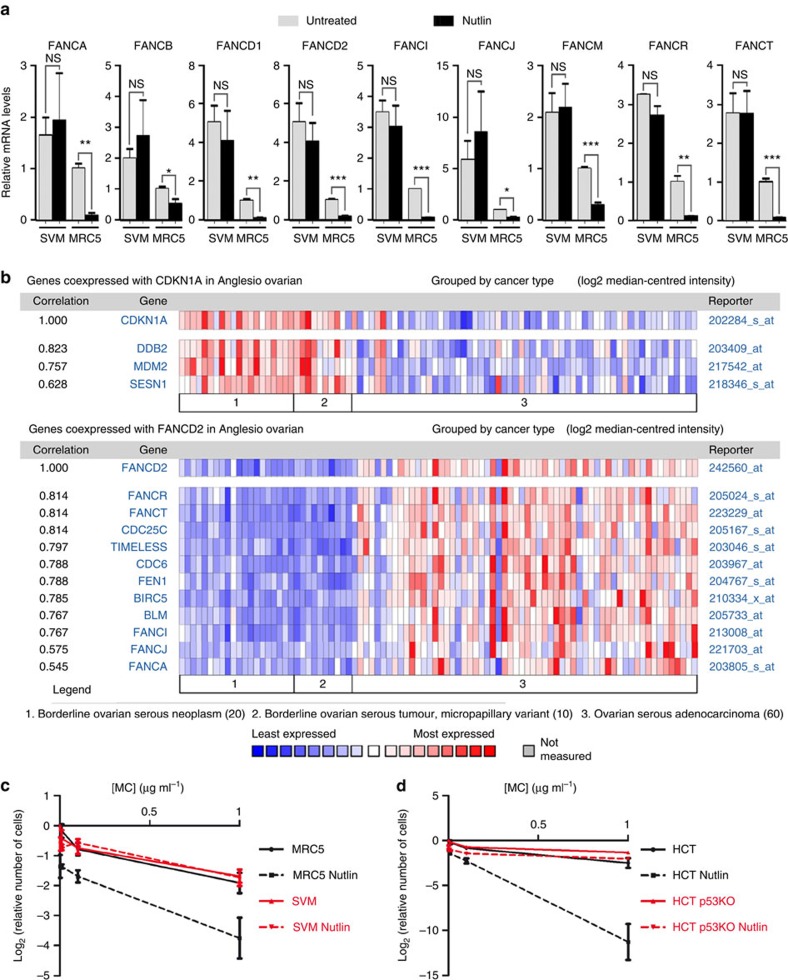
Human p53 also regulates multiple genes of the Fanconi anaemia DNA repair pathway. (**a**) Human p53 activation leads to the downregulation of several *FANC* genes. mRNAs were prepared from human diploid lung fibroblasts (MRC5) and their SV40-transformed derivative cells (SVM), untreated or treated with Nutlin, and mRNAs were quantified using real-time PCR, normalized to control mRNAs, then the amount in untreated MRC5 cells was assigned a value of 1. For each gene, results are from three independent experiments; means+s.e.m. are shown; ***P≤0.001, **P≤0.01, *P≤0.05, NS, not significant by Student's *t*-test. (**b**) In human ovarian cancers, loss of p53 function correlates with an increase in the expression of FANC genes. Analysis of transcriptome data from ref. 47 with the Oncomine software indicates that ovarian serous cancer progression correlates with a decreased expression of p53-transactivated genes (for example, *CDKN1A* and *MDM2*), and an increased expression of several *FANC* genes (*FANCA*, *FANCD2*, *FANCI*, *FANCJ*, *FANCR* and *FANCT*). (**c**) p53 activation sensitizes cells to mitomycin C. MRC5 and SVM cells were treated and analysed as in Fig. 5d. Results from three independent experiments. (**d**) Human cancer cells expressing a WT p53 can be sensitized to mitomycin C by a treatment with Nutlin. Colon carcinoma cells HCT116 (HCT) and their p53^*−/−*^-derivative cells (HCT p53 KO) were treated and analysed as in Fig. 5d. Results from three independent experiments.

We next tested whether the FA genes that were found regulated by murine p53 were similarly regulated in human cells. We compared human primary WT cells with p53-deficient cells, and observed that out of the 12 p53-regulated FA genes identified in mouse cells, 9 are also downregulated upon p53 activation in human MRC5 cells: *FANCA*, *FANCB*, *FANCD1*, *FANCD2*, *FANCI*, *FANCJ*, *FANCM*, *FANCR* and *FANCT* (Fig. 7a). Interestingly, one of these genes, *FANCB*, was recently identified as one of 210 genes most likely to be downregulated by p53 in a E2F4-dependent manner^33^. Furthermore, candidate CDE/CHR motifs could be found for each of these genes (Supplementary Fig. 15a), and the CDE/CHRs in *Fancd2*, *Fanci* and *Fancr* were highly conserved in the human *FANC* homologous genes (Supplementary Fig. 15b). Consistent with this, we next found that human p53 activation leads to increased E2F4 binding at the *FANCD2*, *FANCI* and *FANCR* promoters (Supplementary Fig. 16a), and that mutation of the CDE/CHRs in these promoters abolished their p53-dependent regulation (Supplementary Fig. 16b). The p53-dependent downregulation of *FANC* genes could also be observed in response to DNA damage in MRC5 cells (Supplementary Fig. 17), and we verified that the CDE/CHR motif in *FANCD2* is important for its DNA damage-induced downregulation (Supplementary Fig. 18). In addition, the data mining of a transcriptome-wide analysis were again consistent with our results (Supplementary Fig. 19). *BLM*, *DEK*, *FEN1*, *TIMELESS* and *RECQL4* were also downregulated in human cells upon p53 activation, further indicating an overall conservation of the regulatory pathways identified in murine cells (Supplementary Fig. 20).

Further evidence of this conservation was obtained using the Oncomine software (www.oncomine.org). Tumour samples from the Australian Ovarian Cancer Study revealed that the p53 pathway is functional in low-grade ovarian serous tumours, but frequently lost in high-grade ovarian carcinomas. Evidence for this first came from using a transcriptomic signature of p53 target genes^47^. Formal demonstration was later obtained by *TP53* sequencing, which identified p53 mutations in 0% of low-grade serous tumours^48^ and 96.7% of high-grade carcinomas^49^. We analysed the transcriptome data of Anglesio *et al*.^47^, who characterized 90 ovarian samples from the Australian Ovarian Cancer Study, including 60 high-grade adenocarcinomas. As expected, the expression of genes activated by p53 (*CDKN1A/p21*, *MDM2*, *DDB2* and *SESN1*) was decreased in high-grade tumours. On the opposite *FANCD2*, and other genes known to be repressed by E2F4 in a p53-dependent manner (*BIRC5*, *CDC6* and *CDC25C*), were more expressed in high-grade tumours (Fig. 7b). Increased *FANCD2* expression also correlated with increases in the expression of other FA genes (*FANCA*, *FANCI*, *FANCJ*, *FANCR* and *FANCT*), as well as additional genes regulated by p53 in our experiments (*BLM*, *FEN1* and *TIMELESS*; Fig. 7b). Similar results were obtained when we analysed data from liver cancers (Supplementary Fig. 21) and adrenocortical tumours (Supplementary Fig. 22), providing evidence that human p53 downregulates several genes of the FA pathway in many tissues, and that loss of p53 function leads to an increased expression of *FANC* genes in advanced human cancers.

We next found that Nutlin sensitized human primary WT cells, but not their p53-deficient counterparts, to MC (Fig. 7c). Likewise, the sensitivity to MC of human cancer cells expressing a WT p53 was markedly increased by Nutlin (Fig. 7d), suggesting a potential therapeutic relevance of our findings.

## Discussion

In this report, we further analysed the consequences of a deletion of the p53 carboxy-terminal domain. Our previous analysis indicated that most *p53*^*Δ31/Δ31*^ mice exhibit a full set of features characteristic of DC. At the molecular level, the increased p53 activity in *p53*^*Δ31/Δ31*^ MEFs correlated with the downregulation of four genes implicated in telomere syndromes: *Dkc1*, *Rtel1*, *Terf1* and *Tinf2* (ref. 2). Here we show that several other genes involved in telomere metabolism are downregulated in *p53*^*Δ31/Δ31*^ cells: *Blm*, *Dek*, *Fancd2*, *Fen1*, *Gar1*, *Recql4* and *Timeless*, strengthening the notion that p53 plays a major role in the regulation of telomere metabolism.

Importantly, some of these genes are involved in DNA repair, and we next found *p53*^*Δ31/Δ31*^ cells to exhibit decreased mRNA levels for 11 additional genes mutated in FA, and a reduced capacity to repair DNA interstrand crosslinks. Because DC and FA are both inherited bone marrow failure syndromes in humans, these new findings raised the possibility that an attenuated FA pathway might contribute to the bone marrow failure that affects *p53*^*Δ31/Δ31*^ mice. Importantly, however, mice carrying knocked out alleles of *Fanc* genes exhibit little or no haematological abnormalities in the absence of additional stress^50^ (for example, aldehyde-mediated DNA damage^51^,^52^), whereas aplastic anaemia occurs spontaneously in mouse models of telomere dysfunction (for example, *Pot1b*^*−/−*^ *mTR*^*+/*−^ mice^53^) and in *p53*^*Δ31/Δ31*^ mice^2^. Furthermore, *p53*^*Δ31/Δ31*^ mouse cohorts of mixed genetic backgrounds previously indicated that a gene linked to the *Agouti* locus, on chromosome 2, had an impact on their survival^2^. None of the *Fanc* genes maps on chromosome 2, whereas mRNA levels for *Rtel1*, located 26 cM away from *Agouti*, affected the survival of mutant mice^2^. *Rtel1* encodes a Fancj-like helicase that might participate in DNA repair^54^, but that mainly acts as a dominant regulator of telomere length^55^. Accordingly, *Rtel1* is mutated in telomere syndromes, including severe DC^56^,^57^,^58^ and pulmonary fibrosis^59^. Together, these data indicate that telomere dysfunction most likely plays a predominant role in the aplastic anaemia that affects *p53*^*Δ31/Δ31*^ mice.

Interestingly, aplastic anaemia is not the only clinical trait shared by patients with FA and DC: abnormal skin pigmentation, short stature and testicular hypoplasia may affect patients with either syndrome. Furthermore, telomere dysfunction was reported for at least some patients with FA^28^,^60^, and cells from patients with DC appeared hypersensitive to MC in a few studies 56,61. In fact, although DC and FA are distinct clinical disorders caused by mutations in different genes, their clinical similarities initially led to some confusion^62^,^63^,^64^,^65^, and recent evidence of misdiagnosis can still be found occasionally^66^. As mentioned above, because a defective FA pathway may activate p53 (ref. 46), our results suggest the operation of a positive-feedback loop between p53 and an attenuated FA pathway. Likewise, short telomeres activate p53 (ref. 67), and our data may also suggest a positive-feedback loop between p53 and telomere metabolism. Together, our analyses of *p53*^*Δ31/Δ31*^ mutant cells raise the intriguing possibility that a sustained p53 activation might contribute to the clinical overlap between DC and FA, notably by leading to a concomitant downregulation of genes important for telomere metabolism and genes of the FA DNA repair pathway (Supplementary Fig. 23). Because the p53 pathway is affected by single-nucleotide polymorphisms in many genes including *TP53*, *MDM2*, *MDM4* and *CDKN1A*^68^, we further presume that the strength of the regulatory loops that affect p53, telomere-related and FA genes should vary among humans, and that this might contribute, in patients with identical disease-causing mutations, to the variability in clinical overlap between these syndromes. Independently, our data also provide a rationale for the combination of Nutlin with therapeutic agents inducing DNA interstrand crosslinks, to efficiently kill cancer cells that retain a functional p53 pathway.

## Methods

### Cells and cell culture reagents

MEFs, isolated from 13.5-day embryos, were cultured for ≤6 passages in a 5% CO_2_ and 3% O_2_ incubator, in DMEM Glutamax (Gibco), with 15% fetal bovine serum (FBS; Biowest), 100 μM 2-mercaptoethanol (Millipore), 10 μM non-essential amino acids and penicillin/streptomycin (NEAA/PS, Gibco). BMCs were flushed from femurs and tibias of 3-week-old WT and *p53*^*Δ31/Δ3*1^ mice. The isolation of MEFs and recovery of BMCs were performed according to Institutional Animal Care and Use Committee (IACUC) regulations, as supervised by the Curie Institute's Comité d'éthique en expérimentation animale. NIH-3T3 cells were grown in the same conditions as primary MEFs. Human lung fibroblasts MRC5 and their SV40-transformed derivatives (MRC5 SV2, Sigma) were cultured in a 5% CO_2_ and 3% O_2_ incubator in minimum essential medium (Gibco), completed with 10% FBS, 2 mM L-glutamine (Gibco), 1 mM pyruvate and 10 μM NEAA/PS. Human colon carcinoma cells HCT116 and their derivatives (HCT116 p53 KO, which do not express p53α), kind gifts from Bert Vogelstein (Johns Hopkins University, Baltimore, MD, USA), were grown in a 5% CO_2_ incubator in McCoy's 5A medium with 10% FBS, HEPES and penicilin–streptomycin. Cells were treated for 24 h with 10 μM Nutlin 3a or with 0.5 μg ml^−1^ doxorubicin before PCR with reverse transcription (RT–PCR) or ChIP assays, or 50 nM MC for 48 h before RT–PCR or metaphase spread preparations.

### Quantitative RT–PCR

Total RNA, extracted using Nucleospin RNA II (Macherey-Nagel), was reverse transcribed using Superscript III (Invitrogen). Real-time quantitative PCRs (primer sequences in Supplementary Tables 1 and 2) were performed on ABI PRISM 7500 using Power SYBR Green (Applied Biosystems).

### Western blots

Protein detection by immunoblotting was performed using antibodies raised against Fancd2 (Abcam, ab108928, 1/500 dilution), Fanci (Abcam, ab74332, 1/500), Fancr (Calbiochem, PC130, 1/2,500), E2F4 (Santa Cruz, C-20, 1/200), p53 (Novocastra, CM5, 1/1,000), p21 (Santa Cruz, F-5, 1/250) or actin (Santa Cruz, C-4, 1/5,000) and chemiluminescence revelation was achieved with SuperSignal west dura (Perbio, France). Band quantification was performed using ImageJ, with actin as loading control. Control bands for Fancd2, Fanci and Fancr proteins were obtained using 20 μl lipofectamine 2000 according to the supplier's procedure to transfect NIH-3T3 cells (a 10-cm dish at 60% confluency) with 3 μg of a pCAGGS expression vector (empty pCAGGS vector, pCAGGS-Fancd2, pCAGGS-Fanci or pCAGGS-Fancr—cloning details upon request), then extracting proteins after 24 h. Uncropped scans of the western blots in Figs 1d and 4b, as well as relevant controls, are presented in the Supplementary Figs 2 and 9, respectively.

### ChIP assay

ChIP analysis was performed as described^69^. Briefly, cells were left untreated or treated with Nutlin or doxorubicin for 24 h. Cellular proteins of 10^7^ cells were crosslinked to chromatin with 1% formaldehyde for 10 min at 25 °C. E2F4–DNA complexes were immunoprecipitated from total extracts using an antibody against E2F4 (Santa Cruz, C-20, 30 μg) and 400–500 μg of sonicated chromatin. Rabbit IgG (Abcam) was used for control precipitation. Quantitative PCRs (primer sequences in Supplementary Table 3) were then performed on ABI PRISM 7500.

### Cell cycle assays

Log phase cells, treated or not with Nutlin, were incubated for 24 h, then pulse-labeled for 1 h with bromo-deoxy uridine (BrdU) (10 μM), fixed in 70% ethanol, double stained with fluorescein isothiocyanate anti-BrdU and propidium iodide, and sorted using a LSRII cytometer. Data were analysed using FlowJo.

### Luciferase expression assays

To construct the Luciferase reporter plasmids, we cloned a 2-kb fragment (for *Fancd2*) or 1-kb fragment (for *Fanci*, *Fancr*, *FANCD2*, *FANCI* or *FANCR*) centred around the transcription start site upstream of the firefly luciferase gene in a pGL3-basic vector (Promega), or a variant fragment generated by PCR mutagenesis of the putative CDE/CHR motif (details on request). Next, 10^6^ NIH-3T3 cells were transfected using lipofectamine 2000 by 3 μg of a *Fanc*-luciferase reporter plasmid and 30 ng of renilla luciferase expression plasmid (pGL4.73, Promega) for normalization, and treated or not with 10 μM Nutlin 3a or 0.5 μg ml^−1^ doxorubicin. Transfected cells were incubated for 24 h, then trypsinized, resuspended in 75 μl culture medium with 7.5% FBS and transferred into a well of an optical 96-well plate (Nunc). The dual-glo luciferase assay system (Promega) was used according to the manufacturer's protocol to lyse the cells and read firefly and renilla luciferase signals. Results were normalized, then the average luciferase activity in cells transfected with a WT Promoter and not treated with Nutlin were assigned a value of 1.

### Metaphase spread preparation and analyses

Cells were plated in duplicate, then untreated or treated with 50 nM MC for 48 h, and treated with 0.1 mM nocodazole for 3 h to arrest cells in metaphase. Cells were submitted to hypotonic shock (75 mM KCl), fixed in a (3:1) ethanol/acetic acid solution, dropped onto glass slides and air-dried slides were stained with Giemsa to score for chromosome aberrations. To analyse sister chromatid exchanges, cells plated in duplicate and treated or not with MC were, 1 h after plating, treated with 10 μM (BrdU 1/3 BrdC) for 48 h, then metaphase spreads were prepared as above. Air-dried slides were stained with 10 μg ml^−1^ Hoescht 33258 for 20 min, submitted to ultraviolet at 365 nm while heated at 55 °C during 30 min, then stained with Giemsa. Images were acquired using a Zeiss Axiophot (X63) microscope.

### Immunofluorescence

Cells were spread onto coverslips, treated or not with Nutlin 10 μM, then MC 0.1 μg ml^−1^ for 1 h, and left to recover for 12 h. Twenty-four hours after Nutlin treatment, cells were fixed and permeabilized. Coverslips were incubated with a Rad51 antibody (Ab-1 Calbiochem) for 1 h at 37 °C in a humid chamber, then with secondary Alexa Fluo anti-rabbit antibody (Invitrogen). Slides were mounted in Vectashield with 0.2 μg ml^−1^ 4,6-diamidino-2-phenylindole. Images were captured on a Zeiss Axioplan2 microscope using equal exposure times for all images.

### Cellular sensitivity to mitomycin C

Cells were seeded into wells of a 96-well plate (500 cells per well, in triplicates). After adhesion, cells were treated or not with Nutlin 2.5 μM for 24 h, then with MC for 48 h at 0, 0.01, 0.1 and 1 μg ml^−1^. Cells were then counted using the CyQUANT kit (Life technologies) and a microplate reader according to the supplier's recommendations.

### Statistical analyses

Differences between two groups were analysed by Student's *t*-test, difference between three groups were analysed by one-way analysis of variance, and values of *P*≤0.05 were considered significant.

## Additional information

**How to cite this article:** Jaber, S. *et al*. p53 downregulates the Fanconi anaemia DNA repair pathway. *Nat. Commun.* 7:11091 doi: 10.1038/ncomms11091 (2016).

## Supplementary Material

Supplementary Information Supplementary Figures 1-23, Supplementary Tables 1-3 and Supplementary References
