# Supplementary material for: p53 downregulates the Fanconi anaemia DNA repair pathway
Source: Nat Commun. 2016 Apr 1;7:11091. doi: 10.1038/ncomms11091 (PMC4821997; doi:10.1038/ncomms11091)
Supplement: Supplementary Information — Supplementary Figures 1-23, Supplementary Tables 1-3 and Supplementary References [file ncomms11091-s1.pdf]

Supplementary Figures

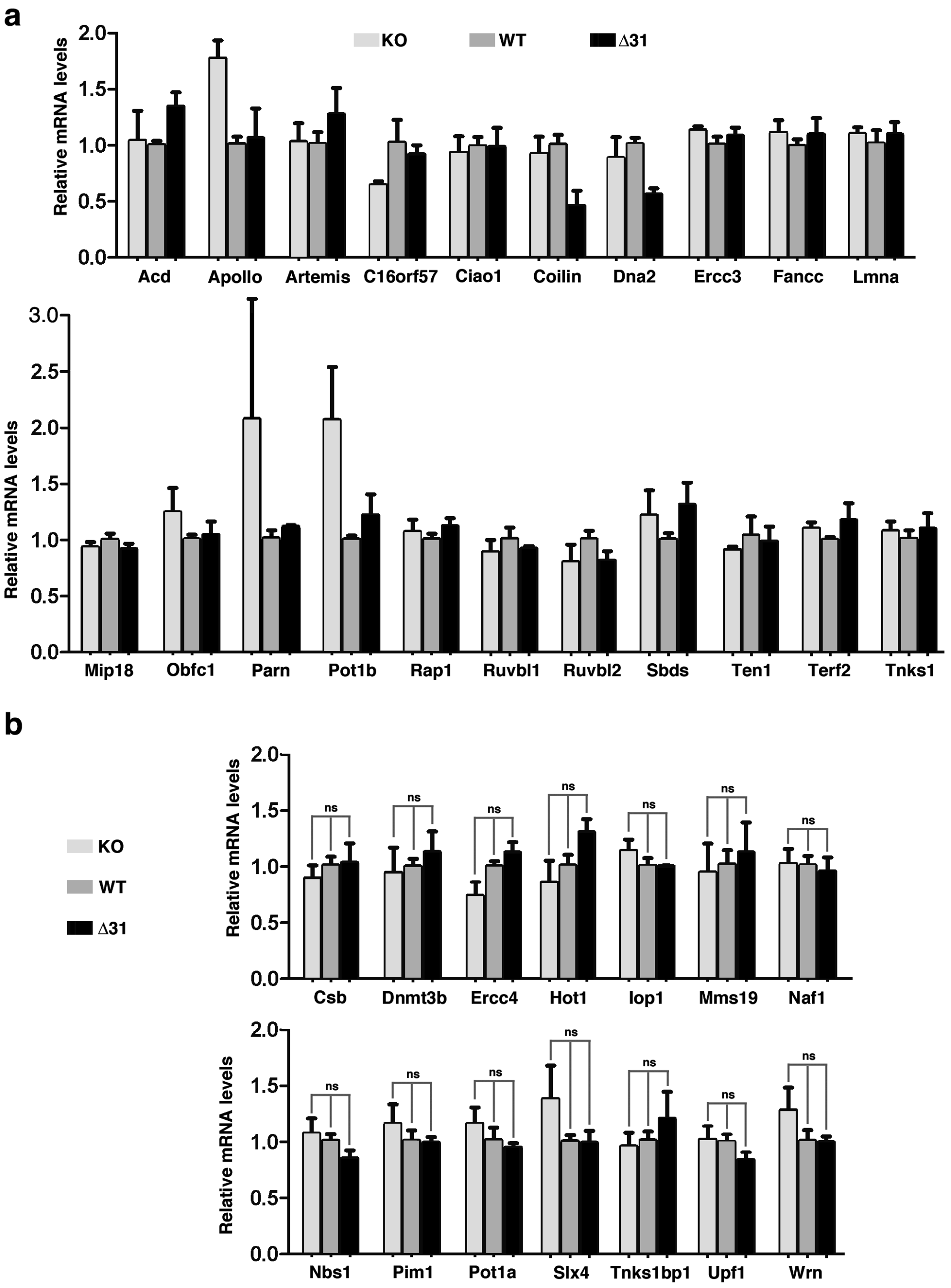

Supplementary Figure 1. Comparison of mRNAs levels for 35 telomere-related genes in *p53*$^{-/-}$, wild-type and *p53*$^{\Delta31/\Delta31}$ cells.

RNAs, prepared from unstressed *p53*$^{-/-}$ (KO), wild-type (WT) and *p53*$^{\Delta31/\Delta31}$ (Δ31) MEFs, were used to compare the expression of 42 genes proposed to impact on telomere metabolism (see Main text for details). Out of 42 genes, 7 exhibited intermediate mean mRNA levels in WT cells compared to *p53*$^{-/-}$ and *p53*$^{\Delta31/\Delta31}$ cells, with significant differences between the means according to one-way ANOVA ; these are shown in Fig. 1a. Here, the 35 genes that did not match these criteria are shown. Results are from ≥ 3 independent experiments. (**a**) For 21 of the tested genes, the mean mRNA levels in WT cells did not fall between those measured in *p53*$^{-/-}$ and *p53*$^{\Delta31/\Delta31}$ cells, making them poor candidates for a p53-dependent regulation. Means + s.e.m. are shown. (**b**) For 14 of the tested genes, the mean mRNA levels in WT cells ranged between those measured in *p53*$^{-/-}$ and *p53*$^{\Delta31/\Delta31}$ cells, but differences between the means were not significant (ns) according to one-way ANOVA, suggesting little or no participation of p53 in the regulation of these genes. Means + s.e.m. are shown, and ns : not significant by ANOVA.

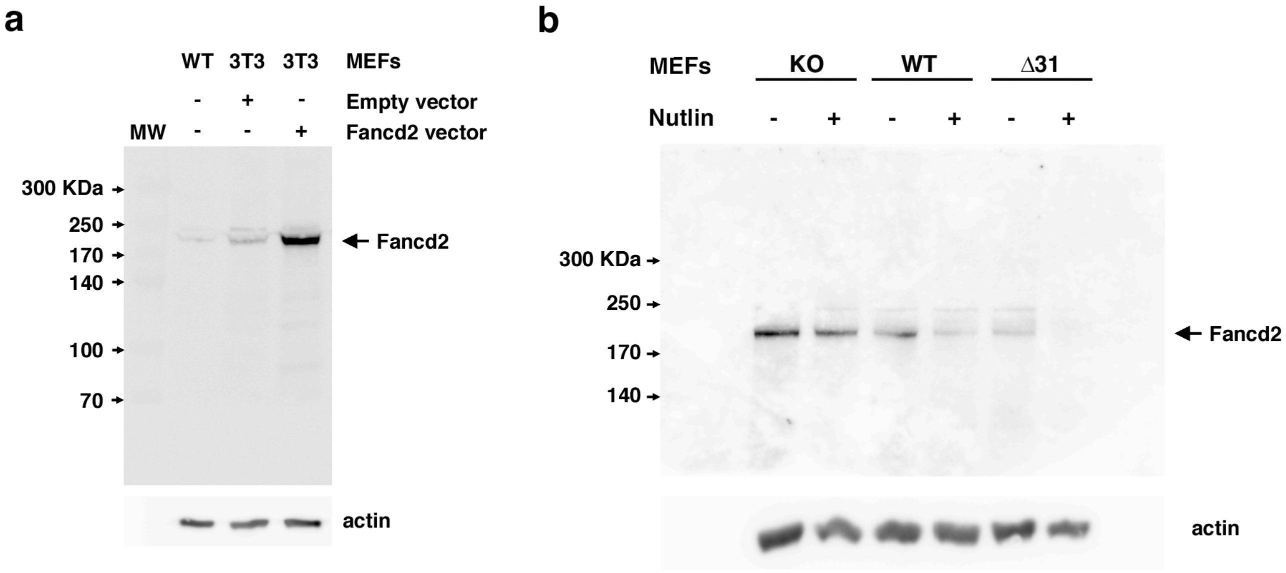

Supplementary Figure 2. p53 activation leads to decreased Fancd2 protein levels.
(**a**) Identification of a Fancd2-specific band in western blots. Protein extracts were prepared from wild-type (WT) MEFs, or NIH3T3 (3T3) cells transfected with either an empty vector or a Fancd2 expression vector, then immunoblotted with antibodies against Fancd2 and actin. The blot demonstrates that the strongest band (at ~200 kDa) detected with the antibody against Fancd2 in WT MEFs corresponds to the Fancd2 protein. (Interestingly, NIH3T3 cells transfected with the empty vector exhibit, compared to WT cells, increased Fancd2 levels, consistant with NIH3T3 cells having a functional but attenuated p53 pathway). (**b**) Protein extracts, prepared from MEFs left untreated or treated with Nutlin (a specific Mdm2 inhibitor [1]) were immunoblotted with antibodies against Fancd2. This western blot is an uncropped version of the blot shown in Fig. 1d. The actin shown in Fig 1d is again shown here for reference.

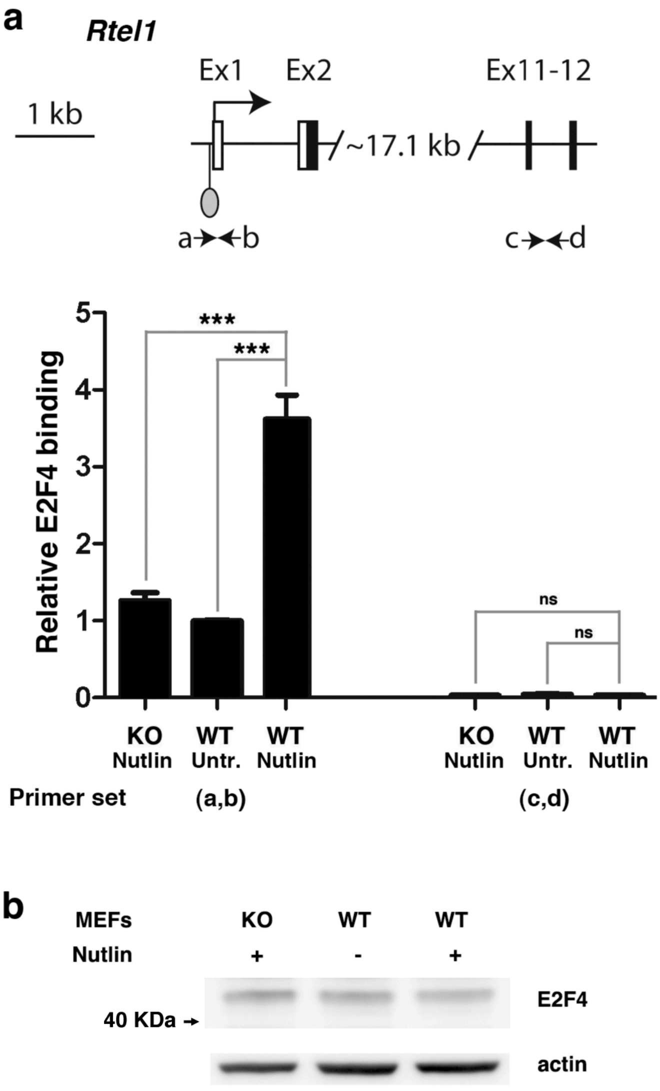

Supplementary Figure 3. A p53/E2F4 – mediated regulation of the *Rtel1* promoter.

(**a**) ChIP assay at the *Rtel1* locus. Chromatin Immmunoprecipitation assays with an E2F4 antibody were first carried out at the *Rtel1* locus, previously demonstrated to be regulated by p53 in a p21-dependent manner [2]. On top, a partial map of the *Rtel1* gene is shown (Ex: exons, black boxes: coding sequences, white boxes: UTRs). Putative E2F4 binding sites were searched for according to Lee *et al.* [3] : they correspond to the presence, within 200 bp, of at least 2 of the 6 following motifs : TTTSSCGC, YSATTGGC, GCGCSGS, CSSYCGCG, CGNAGC or RTTYGAA. ChIP assay for E2F4 binding was performed in Nutlin-treated $p53^{-/-}$ (KO), and untreated (Untr.) or Nutlin-treated wild-type (WT) MEFs, at a putative E2F4 binding site (lollipop) in the *Rtel1* promoter and in a non-binding region from *Rtel1* intron 11, with an antibody against E2F4, or rabbit IgG as a negative control. Immunoprecipitates were quantified using real-time PCR with primer sets a and b, or c and d, respectively. Fold enrichment were normalized to data over an irrelevant region, then E2F4 binding at the Rtel1 promoter in untreated WT cells was given a value of 1. Maximal E2F4 binding at the *Rtel1* promoter was observed in Nutlin-treated WT cells, consistent with a p53-dependent recruitment of E2F4 at this promoter. Data are from 2 independent ChIP experiments, each quantified in triplicates. Means + s.e.m. are shown, and ***P ≤ 0.001, n.s. : not significant by Student's *t* test.(**b**) p53 activation does not lead to E2F4 accumulation. Protein extracts, prepared from Nutlin-treated $p53^{-/-}$, and untreated or Nutlin-treated wild-type MEFs, were immunoblotted with antibodies against E2F4 and actin.

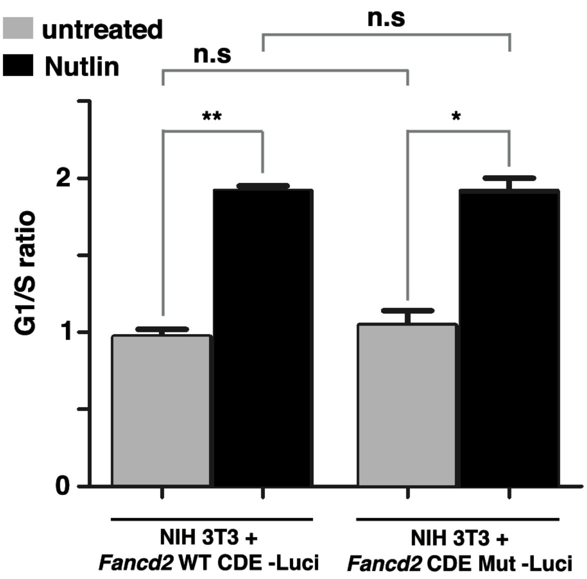

Supplementary Figure 4. NIH 3T3 cells transfected with a WT or a mutant *Fancd2* Promoter-Luciferase reporter plasmid exhibit similar cell cycle kinetics.
NIH 3T3 cells were transfected with a WT or mutant Fancd2 Luciferase reporter plasmid (see Fig. 2c), treated or not with Nutlin, incubated for 24 h, then pulse-labeled with BrdU, double-stained with FITC anti-BrdU and propidium iodide, sorted by FACS, and G1/S ratios were determined. Results from 2 independent experiments, performed simultaneously with the experiments reported in Fig. 2c. Means + s.e.m. are shown, and $**P \leq 0.01$, $*P \leq 0.05$, n.s. : not significant by Student's *t* test.

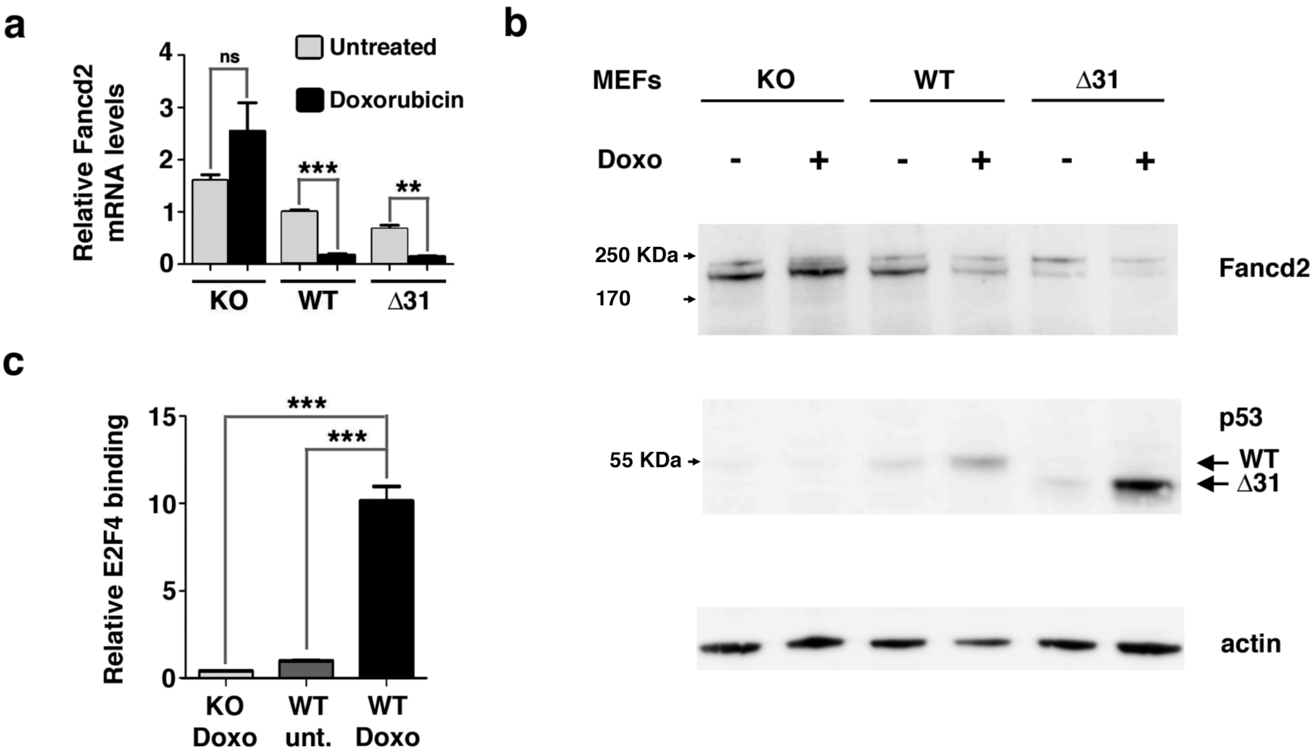

Supplementary Figure 5. The p53-dependent downregulation of *Fancd2* also occurs in response to DNA damage.

(**a**) Upon DNA damage, p53 activation leads to decreased Fancd2 mRNA levels. mRNAs were quantified in $p53^{-/-}$ (KO), WT and $p53^{\Delta 31/\Delta 31}$ (Δ31) MEFs, untreated or treated with 0.5 µM Doxorubicin for 24 hr. Results from 3 independent experiments. (**b**) The DNA damage-dependent activation of p53 leads to decreased Fancd2 protein levels. Protein extracts, prepared from untreated or Doxorubicin-treated (Doxo) MEFs, were immunoblotted with antibodies against Fancd2, p53 and actin. Doxorubicin led to wild-type p53 protein accumulation, and even more so $p53^{\Delta 31}$ accumulation. On the opposite, Fancd2 protein levels were decreased in WT and $p53^{\Delta 31/\Delta 31}$ doxorubicin-treated MEFs (**c**) ChIP assay for E2F4 binding was performed in Doxorubicin-treated $p53^{-/-}$, and untreated (unt.) or Doxorubicin-treated wild-type MEFs, with an antibody against E2F4, or rabbit IgG as a negative control. Immunoprecipitates were quantified in triplicates as described in Fig. 2b. In all figures, means + s.e.m. are shown, and ***P ≤ 0.001, **P ≤ 0.01, n.s. : not significant by Student's *t* test.

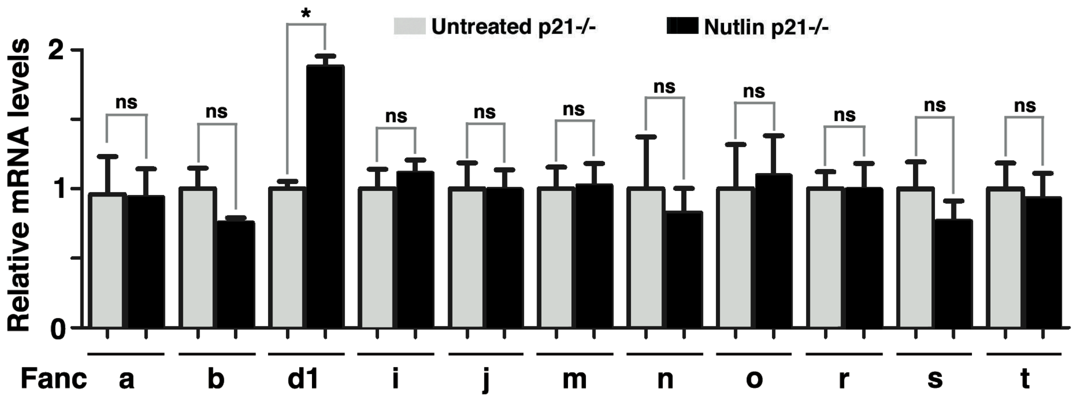

Supplementary Figure 6. The p53-dependent downregulation of *Fanc* genes requires p21.
RNAs prepared from untreated or Nutlin-treated $p21^{-/-}$ MEFs were used to quantify mRNAs for the indicated *Fanc* genes. For each gene, the amount in untreated $p21^{-/-}$ cells was assigned a value of 1. Results from 3 independent experiments. Means + s.e.m. are shown, and *P ≤ 0.05, n.s. : not significant by Student's *t* test.

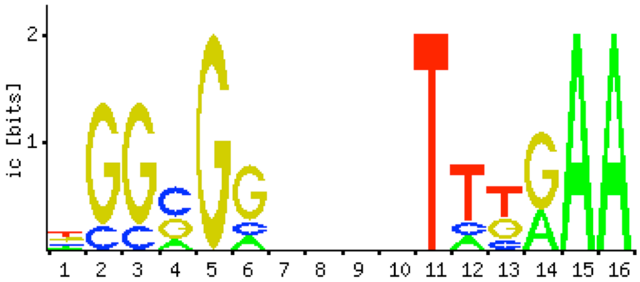

A	1/6	0	0	1/6	0	1/6	1/4	1/4	1/4	1/4	0	1/6	0	1/3	1	1
C	1/6	1/6	1/6	1/2	0	1/6	1/4	1/4	1/4	1/4	0	1/6	1/6	0	0	0
G	1/3	5/6	5/6	1/3	1	2/3	1/4	1/4	1/4	1/4	0	0	1/3	2/3	0	0
T	1/3	0	0	0	0	0	1/4	1/4	1/4	1/4	1	2/3	1/2	0	0	0

	CDE (4bp) CHR	PFM Score
B-myb	TGGCGGGAGATAGGAA	15.66
Cks1	GGGCGGTGTGTTTGAA	17.09
Cyclin B2	CGGCGCGGTATTTGAA	14.39
Plk4	GCGGGAATTTTTCAAA	10.16
Tome-1	TGGAGGGAAGTTTGAA	14.89
Fancd2	AGCGGGAAAGTCGAAA	10.16
Fanca (-13)	CCGCGGCCAATCGGAA	11.69
Fancb (+105)	GGGCGGGAGGTTTGGA	11.73
Fanci (+39)	AGCGGGAATTTTGGAA	12.96
Fancm (-23)	CGGCGGGTTCTTTAAC	9.9
Fancn (-606)	CCGGGCCCTTTTTAAA	10.72
Fanco (+614)	GGAAGGTGGCTTTAAA	9.6
Fancr (+16)	TGGCGGGATTTCCGAA	13.82
Fancs (-113)	GGGGGGGGGTGCTGAA	9.63
Fanct (+484)	CGCAGGCATGTTGAAA	11.13

Supplementary Figure 7. For most *Fanc* genes regulated by p53, a candidate CDE/CHR motif maps less than 500 bp away from the E2F4 binding site identified in chromatin immunoprecipitation assays.

On top, the sequence of 6 functional CDE/CHR motifs (from the mouse genes *Cyclin B2*, *Cks1*, *Plk4*, *B-myb*, *Tome-1* and *Fancd2*) were used to define a positional frequency matrix (PFM), and the 6 motifs were then evaluated with the PFM. Scores from 10.16 (*Plk4*, *Fancd2*) to 17.09 (*Cks1*) were obtained. The mean +/- 2 SD for these scores is 13.7 +/- 5.8. Scores of 10.8 (M – SD) and above are likely to be good CDE/CHR candidates, whereas scores between 7.9 (M - 2SD) and 10.8 would be weaker candidate motifs. Below, the same PFM was used to analyze sequences less than 500 bp away from the center of the amplicon used in ChIP experiments for each gene. Candidate CDE/CHR were found for 9 out of 11 genes, with PFM scores from 9.6 (*Fanco*) to 13.82 (*Fancr*) ; values within a similar range to that observed for the first 6 genes. Number in parentheses are position relative to TSS.

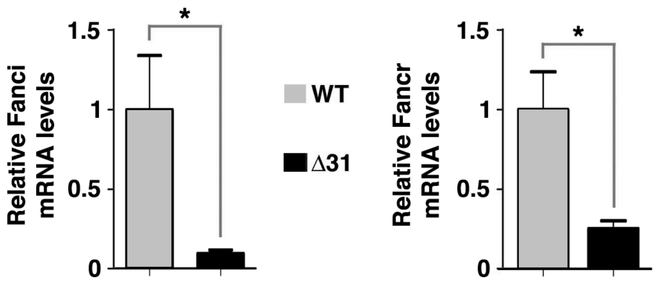

Supplementary Figure 8. The mRNAs for Fanci and Fancr are decreased in bone marrow cells of $p53^{\Delta31/\Delta31}$ **mice.**

Fanci and Fancr mRNAs were quantified from the bone marrow cells of 9 WT and 6 $p53^{\Delta31/\Delta31}$ mice. mRNAs were quantified using real-time PCR, normalized to control mRNAs, then the mean amount in WT cells was assigned a value of 1. Means + s.e.m. are shown, and *P $\leq$ 0.05 by Student's *t* test.

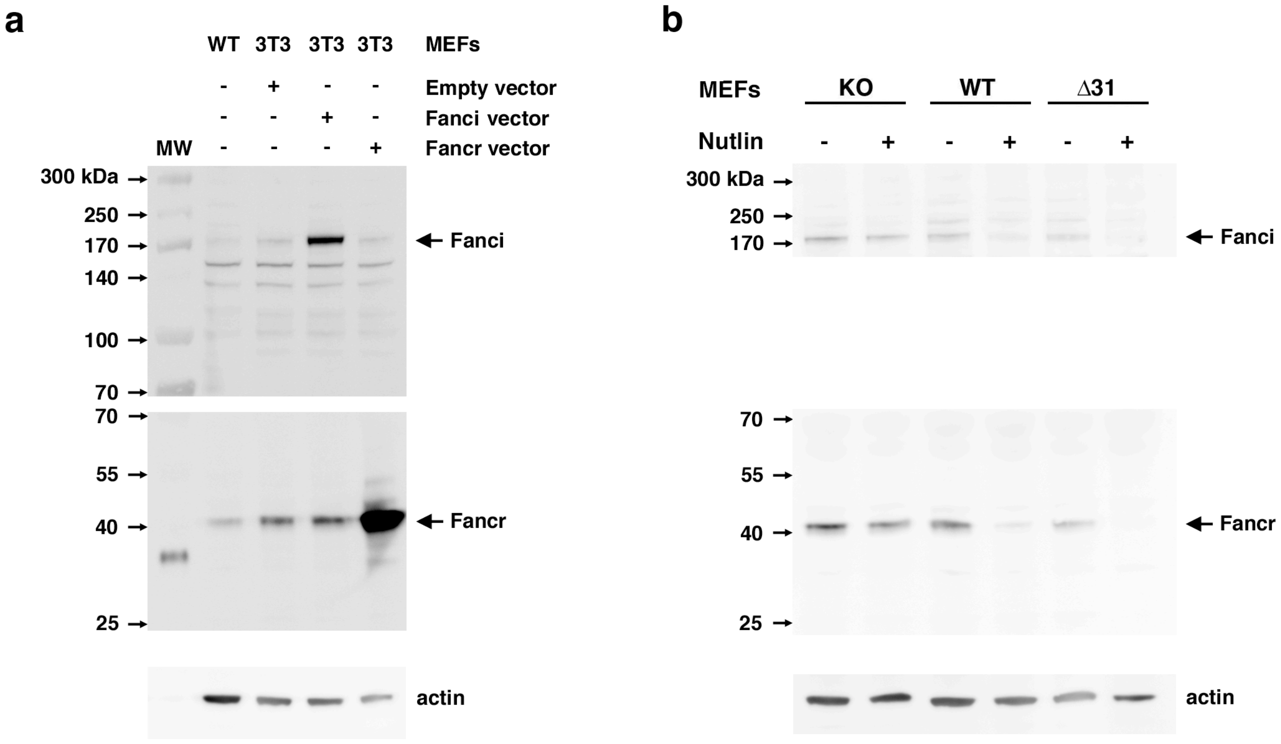

Supplementary Figure 9. p53 activation leads decreased Fanci and Fancr protein levels.
(**a**) Identification of Fanci- and Fancr-specific bands in western blots. Protein extracts were prepared from wild-type (WT) MEFs, or NIH3T3 (3T3) cells transfected with either an empty vector, a Fanci expression vector or a Fancr expression vector, then immunoblotted. The membrane was cut at 70 kDa ; the upper part of the membrane was immunoblotted with an antibody against Fanci, and the lower part with an antibody against Fancr or actin. The upper blot indicates that Fanci is detected as a faint band at ~170 kDa, and the lower blot that Fancr is detected as a strong band at ~40 kDa. In subsequent Fanci western blots, the membranes were cut at around 150 kDa before incubating with the Antibody, to optimize the detection of Fanci specific bands. (**b**) Protein extracts, prepared from untreated or Nutlin-treated MEFs, were immunoblotted with antibodies against Fanci (upper blot), Fancr (middle blot) and actin. The Fanci and Fancr western blots are uncropped versions of the blots shown in Fig. 4b, and the actin already shown in Fig. 4b is shown again for reference.

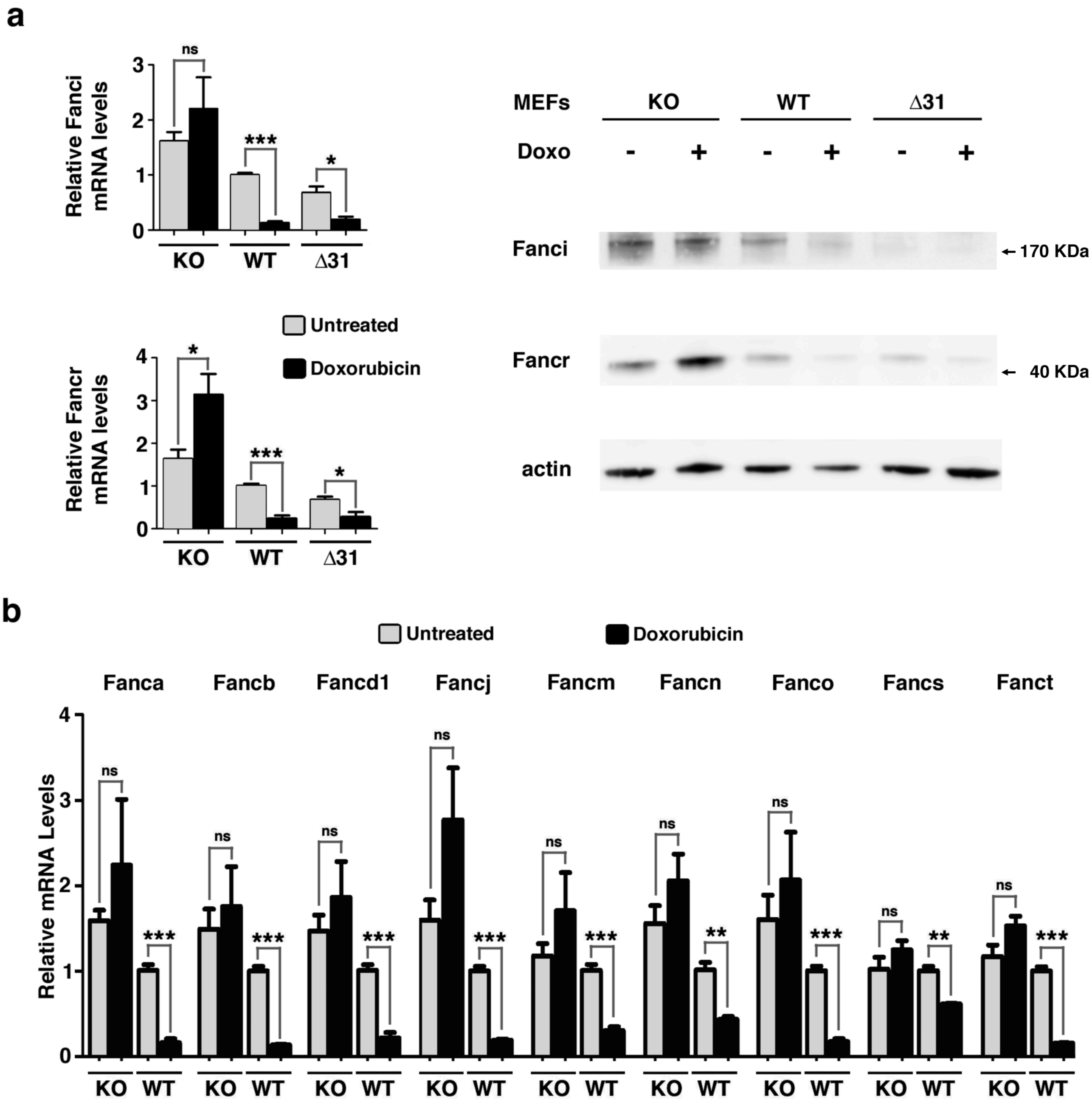

Supplementary Figure 10. The p53-dependent downregulation of several *Fanc* genes also occurs in response to DNA damage.

(**a**) Upon DNA damage, p53 activation leads to decreased Fanci and Fancr mRNA and protein levels. On the left, mRNAs were quantified in *p53*$^{-/-}$, WT and *p53*$^{\Delta31/\Delta31}$ MEFs, untreated or treated with 0.5 µM Doxorubicin for 24 hr. Results from ≥ 3 independent experiments. On the right, protein extracts, prepared from untreated or Doxorubicin-treated MEFs, were immunoblotted with antibodies against Fanci, Fancr and actin. Doxorubicin led to wild-type p53 protein accumulation, and even more so p53$^{\Delta31}$ accumulation (see Supplementary Fig. 5). On the opposite, Fanci and Fancr protein levels were decreased in WT and *p53*$^{\Delta31/\Delta31}$ doxorubicin-treated MEFs. (Note that for actin, this blot is the same as shown in Supplementary Fig. 5). (**b**) Additional *Fanc* genes with a p53-dependent decrease in mRNA levels upon Doxorubicin treatment. mRNAs were quantified in *p53*$^{-/-}$ and WT MEFs, untreated or treated with 0.5 µM Doxorubicin for 24 hr. Results from 2 independent experiments. In all figures, means + s.e.m. are shown, and ***P ≤ 0.001, **P ≤ 0.01, *P ≤ 0.05, n.s. : not significant by Student's *t* test.

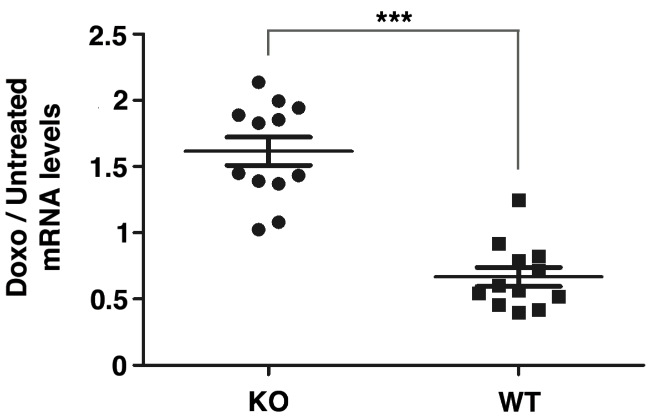

Supplementary Figure 11. Transcriptome data mining supports the conclusion that DNA damage leads to a p53-dependent downregulation of several *Fanc* genes.

From the transcriptome data of Younger *et al.* [4] (Gene Expression Omnibus accession number GSE55727), we extracted the values for the expression of 12 *Fanc* genes *(Fanca*, *Fancb*, *Fancd1*, *Fancd2*, *Fanci*, *Fancj*, *Fancm*, *Fancn*, *Fanco*, *Fancr*, *Fancs*, *Fanct*) in untreated or doxorubicin-treated (Doxo) WT and *p53*$^{-/-}$ (KO) MEFs. For each gene, the ratio of Doxo/untreated mRNA levels was calculated from duplicate (KO) or triplicate (WT) values. The calculated ratios were then plotted, with each point corresponding to a single *Fanc* gene. Although the short treatment with Doxorubicin (6h) most probably accounts for a partial downregulation of *Fanc* genes in WT MEFs, the effects of Doxorubicin were significantly different in *p53*$^{-/-}$ and WT MEFs, with an overall increase in expression for the tested *Fanc* genes in *p53*$^{-/-}$ MEFs, and an overall decrease in WT MEFs. Scatter dot plots and means + s.e.m. are shown, and ***P ≤ 0.001 by Student's *t* test.

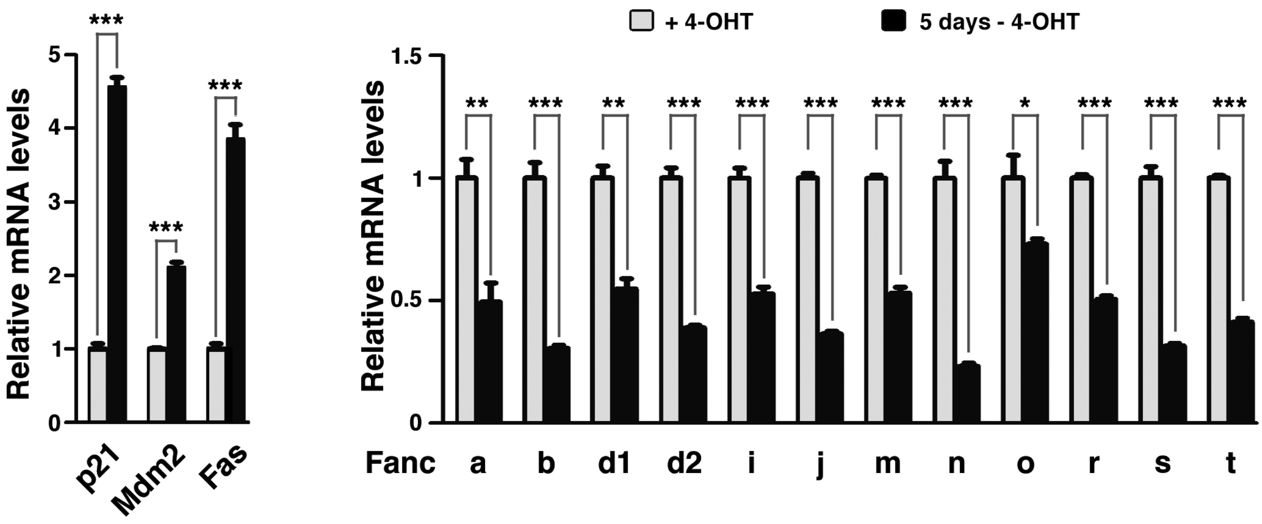

Supplementary Figure 12. Transcriptome data indicate that the myeloid differentiation of hematopoietic cells correlates with p53 activation and the downregulation of 12 *Fanc* genes.
From the transcriptome data of Muntean *et al.* [5] (Gene Expression Omnibus accession number GSE21299), we extracted the robust-multi average (RMA) values for the expression of *Cdkn1A/p21*, *Mdm2*, *Fas*, and 12 *Fanc* genes *(Fanca*, *Fancb*, *Fancd1*, *Fancd2*, *Fanci*, *Fancj*, *Fancm*, *Fancn*, *Fanco*, *Fancr*, *Fancs*, *Fanct*) in Hoxa9-ER expressing cells grown in the presence of tamoxifen (4-OHT), or 5 days after 4-OHT withdrawal (a treatment leading to myeloid differentiation [5]). For each gene, the inverse of Log_2 were calculated from RMA values, and the average in cells with 4-OHT was given a value of 1. Cell differentiation correlated with the transactivation of *p21*, *Mdm2* and *Fas*, and the downregulation of *Fanc* genes. Data from triplicates. Means + s.e.m. are shown, and ***$P \leq 0.001$, **$P \leq 0.01$, *$P \leq 0.05$ by Student's *t* test.

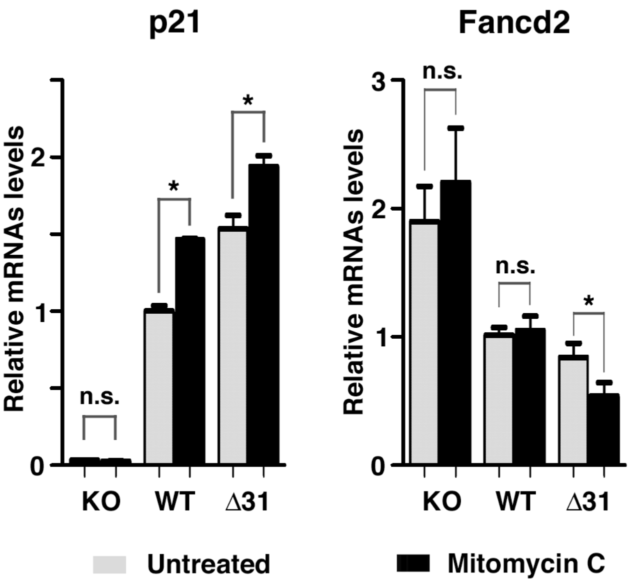

Supplementary Figure 13. Effects of Mitomycin C on WT and *p53*$^{\Delta31/\Delta31}$ MEFs.
mRNAs for p21 and Fancd2 were quantified using real-time PCR, in *p53*$^{-/-}$ (KO), wild-type (WT) and *p53*$^{\Delta31/\Delta31}$ (Δ31) MEFs left untreated or treated with 50 nM Mitomycin C for 48 hr, normalized to control mRNAs, then the amount in unstressed WT cells was assigned a value of 1. Results from ≥ 2 independent experiments. Means + s.e.m. are shown, and *P ≤ 0.05, n.s. : not significant by Student's *t* test.

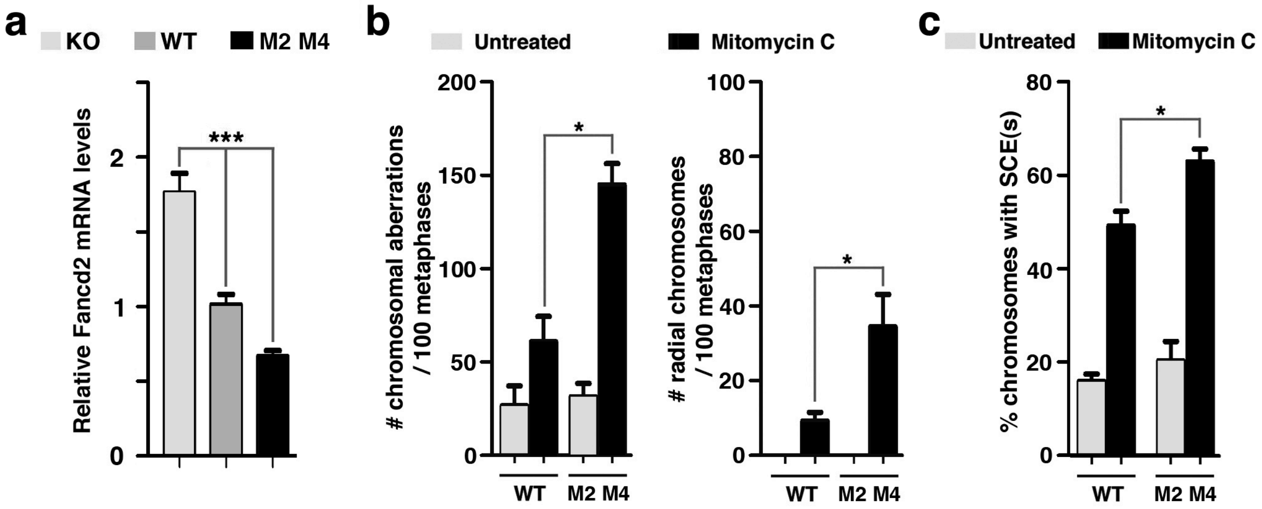

Supplementary Figure 14. ***Mdm2***$^{+/-}$ ***Mdm4***$^{+/\Delta E6}$ **cells exhibit a decreased capacity to repair mitomycin C-induced DNA lesions.**

Because *Mdm2*$^{+/-}$ and *Mdm4*$^{+/\Delta E6}$ mice express lower levels of p53 inhibitors, they exhibit an increased p53 activity while expressing a WT p53 protein [6,7]. *Mdm2*$^{+/-}$ and *Mdm4*$^{+/\Delta E6}$ mice were intercrossed to generate *Mdm2*$^{+/-}$ *Mdm4*$^{+/\Delta E6}$ MEFs. (**a**) mRNAs for Fancd2 were quantified using real-time PCR, in unstressed *p53*$^{-/-}$ (KO), wild-type (WT) and *Mdm2*$^{+/-}$ *Mdm4*$^{+/\Delta E6}$ (M2 M4) MEFs, normalized to control mRNAs, then the amount in WT cells was assigned a value of 1. Results from 3 independent experiments. (**b**) The frequencies of total chromosomal aberrations, or tri- and quadri-radial chromosomes, were determined in wild-type and *Mdm2*$^{+/-}$ *Mdm4*$^{+/\Delta E6}$ MEFs at passage 3, untreated or after treatment with mitomycin C (MC). Results were plotted from 115 (WT untreated), 103 (WT MC-treated), 97 (M2 M4 untreated) and 47 (M2 M4 MC-treated) metaphases. To prevent any potential bias, cell preparations were dropped onto code-labelled slides (to mask the genotypes of cells to be analyzed) and the same metaphases were independently observed by two experimenters. (**c**) A similar procedure was used to determine the percentage of chromosomes presenting sister chromatid exchange(s) (SCEs) after treatment with MC. Results were plotted from an analysis of 2059 (WT untreated), 627 (WT MC-treated), 1788 (M2 M4 untreated) and 647 (M2 M4 MC-treated) chromosomes. In all figures, means + s.e.m. are shown, and ***P ≤ 0.001, *P ≤ 0.05 by ANOVA or Student's *t* tests.

a

	CDE (4bp) CHR	PFM Score
FANCA (-384)	GGGCGGTGTTTCGGCA	9.33
FANCB (+15)	TGGCGGGAGGTTTGGA	11.73
FANCD1 (-438)	GGGCTGTTATTTGAAA	10.20
FANCD2 (+21)	GGCGGGAAAGTCGAAA	11.02
FANCI (+57)	AGCGGGCTTTTTGGAA	12.96
FANCJ (-377)	AGCTGGGTAATTTAAA	8.73
FANCM (+6)	CCGATGGGGATCGGAA	4.91
FANCR (+8)	TGGCGGGAATTCTGAA	15.25
FANCT (-550)	GGGTGGCAGTTTGGAA	12.13

b

	CDE (4bp) CHR
Fancd2 (+23)	AGCGGGAAAGTCGAAA
FANCD2 (+21)	GGCGGGAAAGTCGAAA
Fanci (+39)	AGCGGGAATTTTGGAA
FANCI (+57)	AGCGGGCTTTTTGGAA
Fancr (+16)	TGGCGGGATTTCCGAA
FANCR (+8)	TGGCGGGAATTCTGAA

Supplementary Figure 15. Candidate CDE/CHRs in human *FANC* genes downregulated by p53.
(a) Candidate CDE/CHRs are found close to the TSS of the 9 *FANC* genes downregulated by p53. Sequences surrounding the TSS of each indicated *FANC* gene were analyzed as described in Supplementary Fig. 5. Numbers in parentheses indicate positions relative to the TSS. **(b)** The CDE/CHR motifs identified in the murine *Fanc* genes are well conserved in their human homologs. A strong conservation is observed between the 3 murine CDE/CHRs that we tested in luciferase assays (*Fancd2*, *Fanci*, *Fancr*) and their human counterparts (*FANCD2*, *FANCI*, *FANCR*).

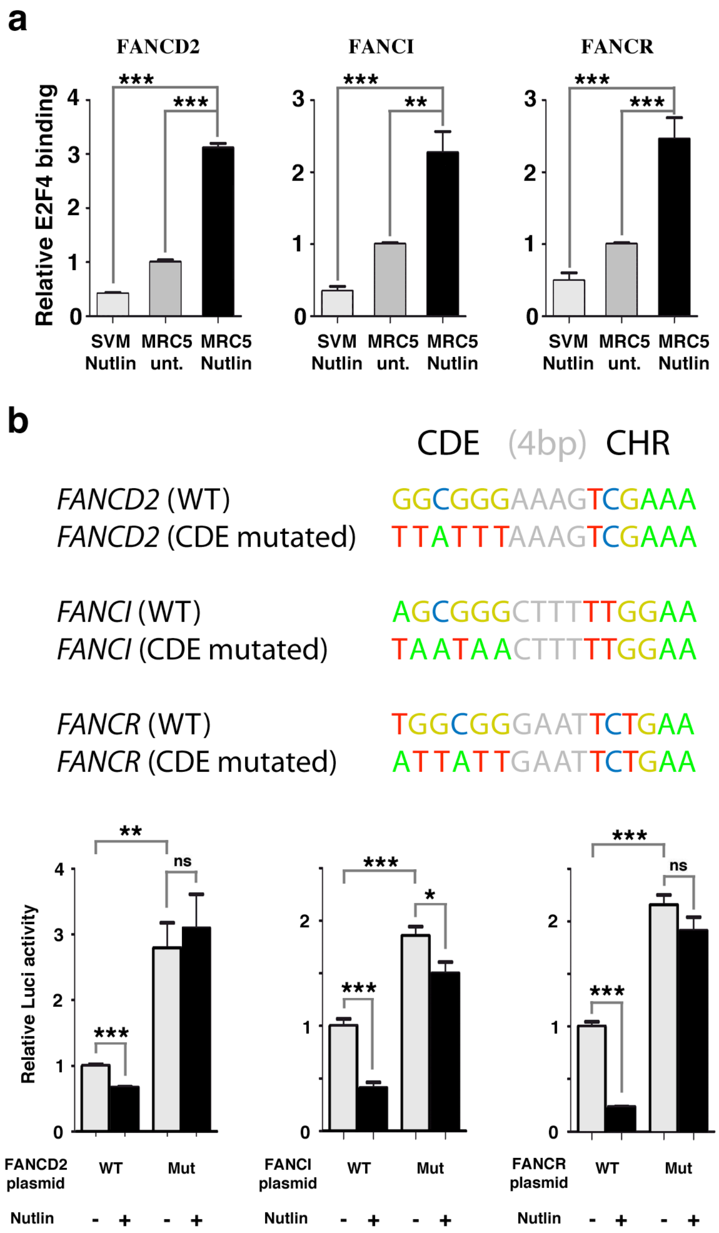

Supplementary Figure 16. The p53-mediated downregulation of *FANCD2*, *FANCI* and *FANCR* relies on CDE/CHR motifs.

(a) Increased E2F4 binding at the *FANCD2*, *FANCI* and *FANCR* promoters upon p53 activation. ChIP assay for E2F4 binding was performed in Nutlin-treated SV40-MRC5, and untreated or Nutlin-treated MRC5 human fibroblasts, with an antibody against E2F4, or rabbit IgG as a negative control. For each gene, immunoprecipitates were quantified using real-time PCR with primers flanking the candidate CDE/CHR, fold enrichment were normalized to data over an irrelevant region, then E2F4 binding at the promoter in untreated MRC5 cells was given a value of 1. Data are from 2 independent ChIP experiments, each quantified in triplicates. (**b**) The p53-dependent regulation of *FANCD2*, *FANCI* and *FANCR* occurs via a CDE/CHR motif. On top, the sequences for the candidate CDE/CHR motifs and their mutated couterparts are shown. Below, for each gene a 1 kb fragment centered around the TSS site, containing a WT or mutant CDE/CHR, was cloned upstream of a Luciferase gene and transfected into NIH-3T3 cells, treated or not with Nutlin, then Luciferase activity was measured after 24 hours. Nutlin led to decreased luciferase activity with the construct containing a WT CDE/CHR motif. Mutation of the putative CDE site increased Luciferase basal expression, and fully (*FANCD2*, *FANCR*) or partially (*FANCI*) abrogated the effect of Nutlin. Results from 2 independent experiments, each in duplicate. In all figures, means + s.e.m. are shown, and ***P ≤ 0.001, **P ≤ 0.01, *P ≤ 0.05, n.s. : not significant by Student's *t* test.

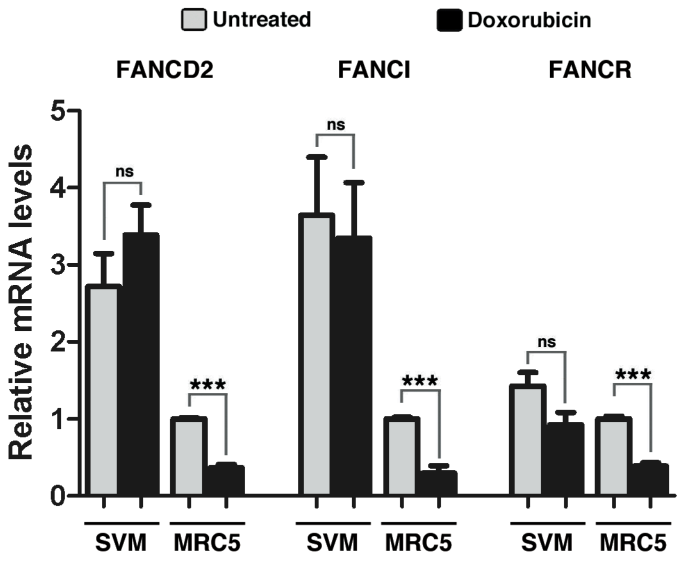

Supplementary Figure 17. p53 downregulates several *FANC* genes in response to DNA damage. mRNAs were prepared from human diploid lung fibroblasts (MRC5) and their SV40-transformed derivative cells (SVM), untreated or treated with Doxorubicin for 24 h, and the mRNAs for the 3 indicated *FANC* genes were quantified using real-time PCR, normalized to control mRNAs, then the amount in untreated MRC5 cells was assigned a value of 1. For each gene, results are from 3 independent experiments. Means + s.e.m. are shown, and ***P ≤ 0.001, n.s. : not significant by Student's *t* test.

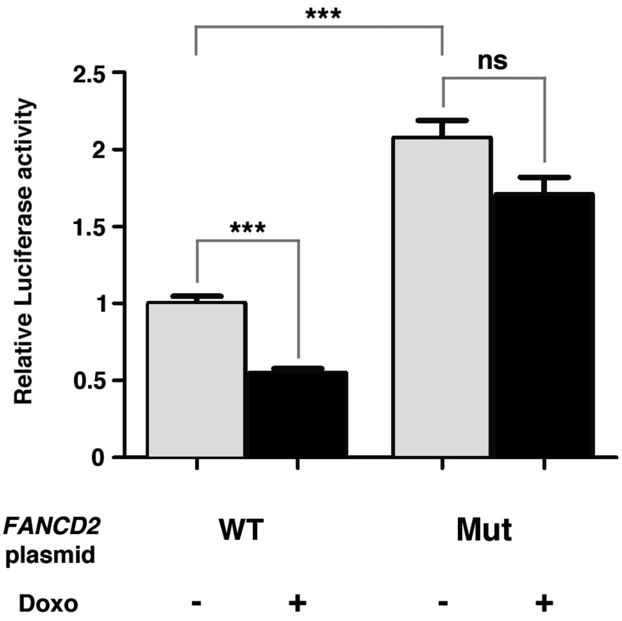

Supplementary Figure 18. The CDE/CHR motif in *FANCD2* is important for the downregulation of its expression in response to DNA damage.

The Luciferase reporter plasmids containing a *FANCD2* promoter region with a WT or mutant CDE/CHR motif (for details, see Supplementary Fig. 16) were transfected into NIH-3T3 cells, treated or not with Doxorubicin, then Luciferase activity was measured after 24 hours. Results from 2 independent experiments, each in duplicate. Means + s.e.m. are shown, and ***P ≤ 0.001, n.s. : not significant by Student's *t* test.

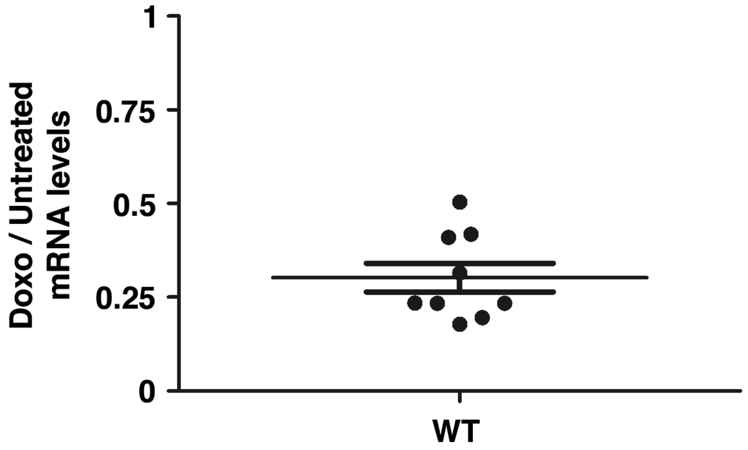

Supplementary Figure 19. Transcriptome data mining supports the conclusion that DNA damage leads to a p53-dependent downregulation of several *FANC* genes.

From the transcriptome data of Younger *et al.* [4] (Gene Expression Omnibus accession number GSE55727), we extracted the values for the expression of 9 *FANC* genes *(FANCA*, *FANCB*, *FANCD1*, *FANCD2*, *FANCI*, *FANCJ*, *FANCM*, *FANCR*, *FANCT*) in untreated or doxorubicin-treated (Doxo) WT human fibroblasts. For each gene, the ratio of Doxo/untreated mRNA levels was calculated from 2 cell lines, each analyzed in duplicates. The calculated ratios were then plotted, with each point corresponding to a single *FANC* gene. Scatter dot plot and mean + s.e.m. are shown. Treatment with Doxorubicin, for 12 h in these experiments, led to a ≈ 2-5 fold decrease in *FANC* mRNA expression.

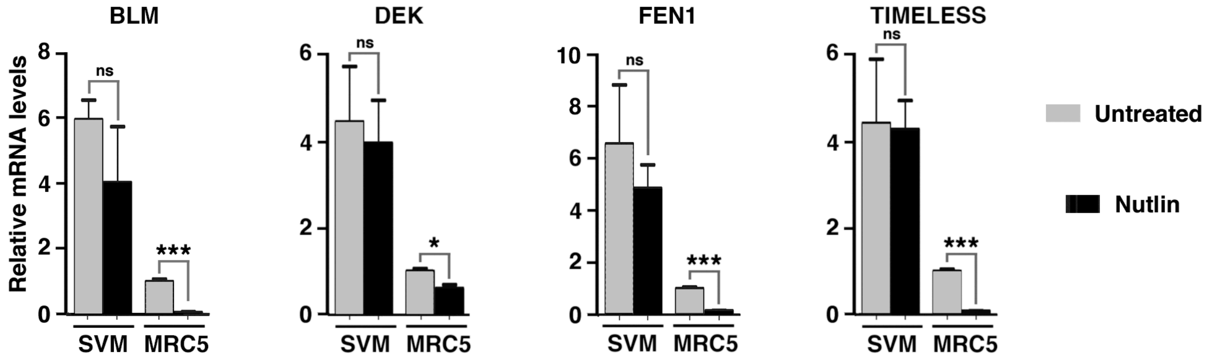

Supplementary Figure 20. p53 activation leads to the downregulation of *BLM*, *DEK, FEN1* and *TIMELESS* in human cells.

(b) mRNAs were prepared from human diploid lung fibroblasts (MRC5) and their SV40-transformed derivative cells (SVM), untreated or treated with Nutlin, and mRNAs were quantified using real-time PCR, normalized to control mRNAs, then the amount in untreated MRC5 cells was assigned a value of 1. For each gene, results are from 3 independent experiments. In addition to those 4 genes, *RECQL4* was already reported to be downregulated in human cells upon p53 activation [8]. Means + s.e.m. are shown, and ***P ≤ 0.001, *P ≤ 0.05, n.s. : not significant by Student's *t* test.

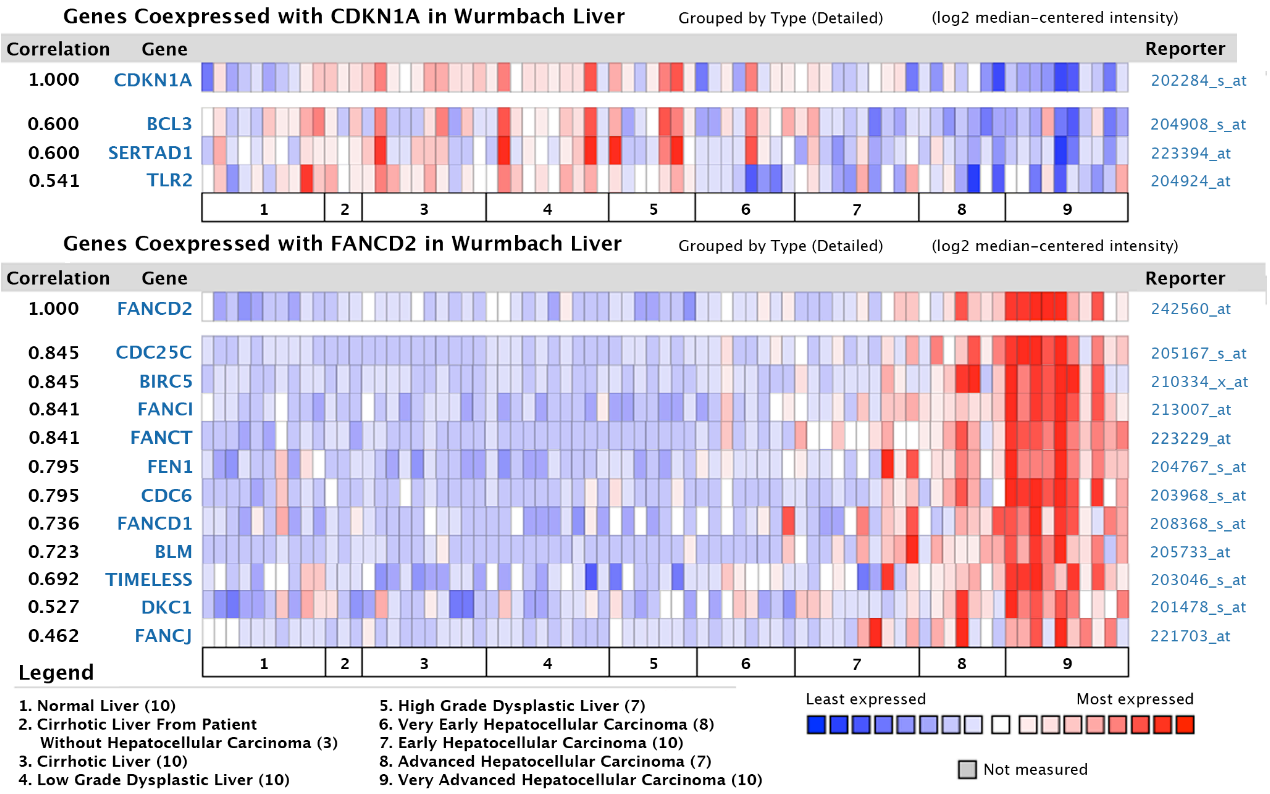

Supplementary Figure 21. Liver cancer progression correlates with a decreased expression of p53-transactivated genes and an increased expression of several *FANC* genes.

TP53 mutations are known to arise at later steps of hepatocarcinogenesis [9]. Analysis of transcriptome data from Wurmbach *et al.* [10] with the Oncomine software indicates that liver cancer progression correlates with a decreased expression of p53-transactivated genes (e.g. *CDKN1A, BCL3*), and an increased expression of several *FANC* genes (*FANCD1*, *FANCD2*, *FANCI*, *FANCJ, FANCT*), telomere related genes (e.g. *DKC1*), and genes already known to be repressed by E2F4 in a p53-dependent manner (e.g. *BIRC5*).

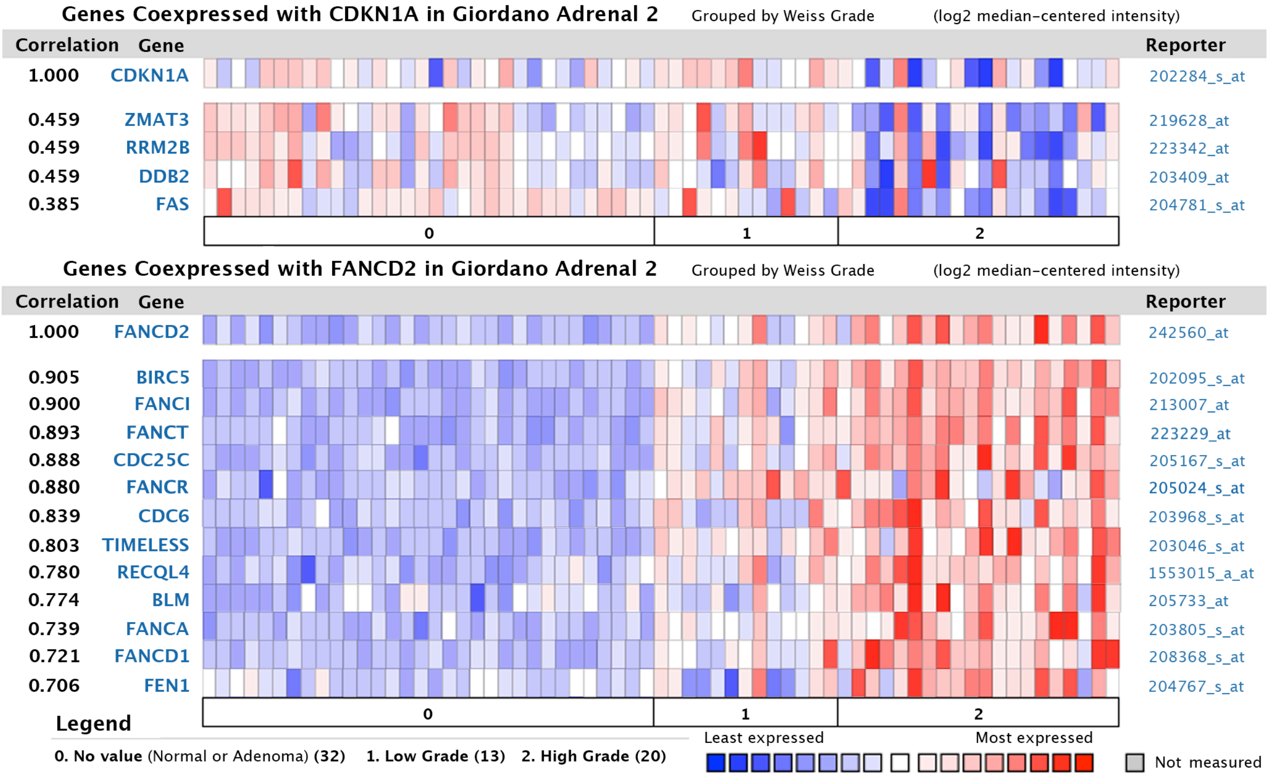

Supplementary Figure 22. The progression of adrenocortical carcinomas correlates with a decreased expression of p53-transactivated genes and an increased expression of *FANC* genes. *TP53* mutations are known to occur in aggressive adrenocortical tumors [11]. Analysis of transcriptome data from Giordano *et al.* [12] with the Oncomine software similarly indicates that the progression of adrenocortical tumors correlates with a decreased expression of p53-transactivated genes (e.g. *CDKN1A, FAS*), and an increased expression of several *FANC* genes (*FANCA*, *FANCD1*, *FANCD2*, *FANCI, FANCR, FANCT*).

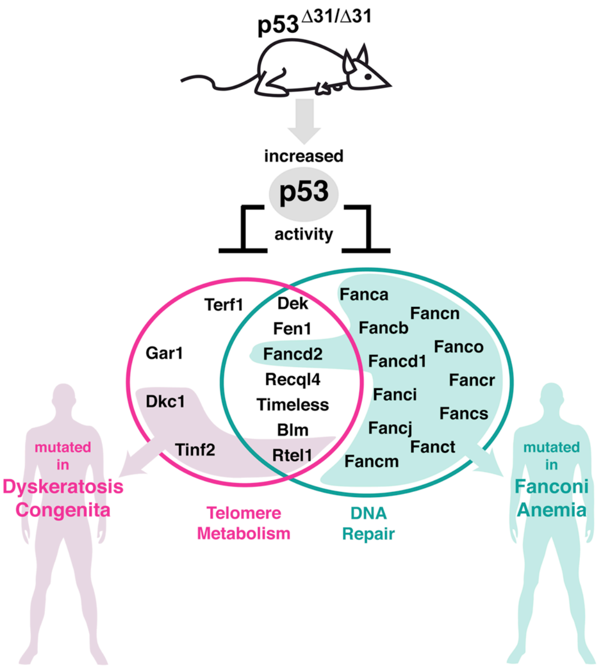

Supplementary Figure 23. ***p53*** $^{\Delta 31/\Delta 31}$ **MEFs reveal the impact of increased p53 activity on telomere metabolism and / or DNA repair.**
This cartoon summarizes data from the present study and Supplementary ref. 2.

Supplementary Tables

Gene	Forward Primer (5'-3')	Reverse Primer (5'-3')
Acd/Tpp1	GAGCTGGTGCAGGCTGTGT	CATCCTCCAGGGTTAGGTACTTTC
Apollo/Snm1b	AATTGGGCAAGAAACCATGACT	TGACAGAACCAGGGCAGTGA
Arp	ATCTGCTGCATCTGCTTG	CGACCTGGAAGTCCAACTAC
Artemis/Snm1c	AAACAAAACTGCACTCCACACAA	TCCTCTCTCCAAACCACATGGT
Blm	TGTGATTCATGCATCTCTTCCTAAA	CAGCTCGGCCGGATTCT
C16orf57/Mpn1/Usb1	GGACAGTGCCTGCAGGAACT	CGTAACAGCATCTCAGAGTCTTCAA
Ciao1	GCTTCCACACCAGGACCATT	AGCCAAGGCCCCTGTCA
Coilin	AACCTGAGAAGCCATCAGATCTG	CAACCCTGTGTCTGCTTGTGA
Csb/Ercc6	AGCCGCTGATTACAAAATACAATG	CCCGTGTGGTCAGAAGAAAGA
Dek	CAGCAGTGACAGCGCAGAGT	GGCAAAGCAGAACTGGACAGT
Dna2	GACAGCAGCTGCGGATCAT	CCTCAACCATCCCAACAGAAG
Dnmt3b	CACTCTGCTCACCCACATGCT	AGACGATCTAGGTGGAGCTTCCT
Ercc3/Xpb	CGGCGGGTTCTGACTATTGT	CAAGGGTTGCAGTCAAACCA
Ercc4/Fancq/Xpf	GGGACGGCCCGAAGAG	TTGTTTGGAGCGGCTTTTG
Fanca	CTGCCCTGCGCAACTGT	TGGAATGTGAAACACGTACCTTTT
Fancb	CCCTGAGGAAAACTTACCAGACAAT	TGGCACCATGTCTGCTCTACA
Fancc	CGGCCGCGGGAAAA	GCACAGCCTCACGGAACTTT
Fancd1/Brca2	TCCCCCCTACCATCAGTTTG	CAGTGGTAGAGTTTGACTTCGTTCTT
Fancd2	TGGGTACTCTCAAAAACCGAGACT	GGAAGAGGGATCCTGGGAAA
Fance	GCTGCTGCGAAGGGATGT	GACAGCTTCTGGTCAATGGAGAA
Fancf	AGGACATGCCCTGGGAAGA	CTGGGCATGGCACAAGCT
Fancg	TGCTCAACCGTACCTCGTCTCT	TCCTGGCATTTTCCCACAAG
Fanci	TCGTGGTGGCCAAAGTGTT	CCAAAGGTGGAATTTCTTGAAGA
Fancj/Bach1/Brip1	TCTGCTAAACAAGGAAACAACCAA	TGTCCCCCGTGATCTTTCTT
Fancl	AATCTGTTATGCCCGTCACC	TCTGTCTGCTGGTGCTCAAC
Fancm	GGCAGAACGTGTCCAAGATTG	GCGGAGCCTTTTCTGATGTT
Fancn/Palb2	CTGGTGATGACAGTGAAAAGCAA	CAGGCCAAGCATAGCTTTTATATCT
Fanco/Rad51c	CGGTACATCTGTGGCAAACG	GGGTATGCTCTTGCTCGAGAA
Fancr/Rad51	AGTGGAGGCTGTTGCTTATGC	CTTTGGCTTCACTAATTCCCTTAATATT
Fancs/Brca1	CCAGCCGGGCACCTTAG	ATCCGCTGCAGGTTCAGTGT
Fanct/Ube2t	TGAAGAAGGAACTGCACATGCTA	TCCTGCCAGCACGTGATG
Fen1	CGCAAAGTGGCCATCGAT	CCACCCTGACGAACAGCAAT
Gar1/Nola1	CGGTCGTGGAGGCTTTAATAAA	TCCTAACAAGACGACACGTTCTG
Hot1	AGGACCCTGAATGGAGACAAAC	CGTCGAAGCCGGAAGGTT
Iop1/Narfl	CACATTGAAGACGATGGCAGTT	TGGTGAGACCTCAGGGTCCTT
Lmna/Progerin	GGCCCTGGGAGAGGCTAAG	CATCCACTCGCCTCAGCAT
Mip18	AGCGACCCAGAGAGCACAGT	TGCTGCAGTGTGGGATGGT
Mms19	TTCTAACCGCCGAGACTAAAGG	AGGCCACTAGTGTGGTTCAGAAA
Naf1	GATTCAAGGCCGCAAAAAAC	CATGCACTTCACCAAAATCTTCA
Nbs1	CGGACCCTCACTGTGGAAAA	CCTGTCCAGGGTCGCATT
Obfc1/Stn1	CAGCAGAAGATCTACCACATCATTAAG	TGGCAGCCCTTCTCCATATG
Parn	CCTGGTGAATGTTGGCAAGTC	TCTCCTGGCGTGGTTCACA
Pim1	GCCTGTCCCTGAGACCATCA	ATGGATGGTTCCGGATTTCTT
Pot1a	TGGCTAGAGTGCCTCCTCAAG	GGTAGCAAATTCGTCGCTCTGT
Pot1b	CCTGGGCAAAGCACAAGTG	CCTGGTGCCATCCCATACC
Ppia	CAGTGCTCAGAGCTCGAAAGTTT	TCTCCTTCGAGCTGTTTGCA
Rap1/Terf2ip	GAAGGACGATGACGACACGAA	GAGCAACGTTCTGAGCTCCAA
Recql4	GGTGCTGGAAAGTCTCTGTGCTA	GGGCTTCGCTGGGCATA
Ruvbl1	AGGCAAGGCAGGTGTGACA	GGACGTACTCTTCAGCTTCAAGGT
Ruvbl2	TGCTGGAGATGATCCGAGAAG	TGGCTGGCCTGCAATGA
Sbds	ACGACCAGACTGAAATCTGCAA	TGCCGTTCTTTATCTGACACTTG
Slx4/Fancp	AAACCGTTCCTCACCACCAA	TCCTCACCGCACACTGCTT
Ten1	AAGGAAGCACACTGCGAACA	GGGAGCGTGCCATGTCAT
Terf2	GGTGAAGACAGGTCATCCAGTTT	ACCCACTCGCTTTCTTCTATGG
Timeless	CGTCTCCTTTGGGCAATTCA	CTGGACAGGAAGAGGAGCAAGT
Tnks1	GCCGGCCGAAAGTCTTCT	CAACATCCTTCCTTCCAAAACC
Tnks1bp1	CTGAGATCCTTGATAGTGCCATGT	GCCCCCGCTTACGTCCTA
Upf1	GGGCCCAGTGGTGATGTG	CTCGAAGAGCGATTGTGACAGT
Wrn	ACGGTGTCTCTGAAGGCAAAG	TGACAGAAATGTTTGATGACTTCCA

Supplementary Table 1. DNA sequences for primers used in mouse RT-qPCR experiments. The primers for *Arp* and *Ppia* were used for normalizations.

Gene	Forward Primer (5’-3’)	Reverse Primer (5’-3’)
BLM	GAGGCAGCAGCTGAATCATCT	GCTTCTGGGTTTCGTGTTATGC
DEK	CATTGCCGAAATCTAAAAAAACTTG	CCTTGCCATTCCAGAACTGTTC
FANCA	CCGAGAGGTGTTGAAAGAGGAA	GGCATGATGCAGGAGAAGGA
FANCB	CAAATCCTTTCCCAGCACCAT	GACCCTTTTTGCTTCCAATCC
FANCD1/BRCA2	ACCATATTTACCATCACGTGCACTA	TGCACCATCTTGCAAAGCA
FANCD2	AGACTGTCAAAATCTGAGGATAAAGAGA	TGGTTGCTTCCTGGTTTTGG
FANCI	CAGGCAACCCTACCAAATCAG	GCAGAGTTCCCAGTTGCATGA
FANCJ/BACH1/BRIP1	AACAGTTGACATCTACCCAAGAAATCT	TCAGTGGAATGCAGCACCAA
FANCM	TGGAGACTGTGGCAAGATCATC	GCGGCATCGATCTGAGTGA
FANCN/PALB2	CCGGTTGTAAAGAGCCATGTATC	ATCCAGAGCTTTCCAAAGAGAAAC
FANCO/RAD51C	GTGGCAGGTGAAGCAGTTTTTA	GCAAGGTCTACCACTCTATCAACCA
FANCR/RAD51	TGGGAGATGCCAAAGACTGAA	AGGCTGCAGCACTTAAGGTTTT
FANCS/BRCA1	TGGTGCGATCATGAGGCTTA	TGAGGTGATAGGATCGCTTGAG
FANCT/UBE2T	GGGATCATGCAGAGAGCTTCA	GGGTGGCTCTGTGGCTAACA
FEN1	CGGGCTGTGGACCTCATC	TCAAGTCGCCGCACGAT
PPIA	CAAATGCTGGACCCAACACA	TGCCATCCAACCACTCAGTCT
RPLP0	CTTGTCTGTGGAGACGGATTACAC	TACGCCAAGAAGGCCTTGA
TIMELESS	CTGGCCCATGACCTCAAAAT	CGATTGAAGAGGCAGAAGACTGA

Supplementary Table 2. DNA sequences for primers used in human RT-qPCR experiments. The primers for *PPIA* and *RPLP0* were used for normalizations.

**Mouse Gene**	**Forward Primer (5'-3')**	**Reverse Primer (5'-3')**
Fanca	CAGACTCCGGGACAAGTACTACAA	GAGGGCGGACCCTTGCT
Fancb	CCTCGGCCATGGTCACA	GAAGGAGCGACGGGTACAAC
Fancd1/Brca2	ACATGCTAGCCGGGAACAG	AGTGCCACCGGGAAGTCA
Fancd2	CGTCAGAGCGCCGTTCTT	GCCAGTTGCTTCCCTTCGT
Fanci	TGGGAAGAGAGGCCATTGAC	TCGACAGTGCACGTCTTGAGT
Fancj/Bach1/Brip1	TCGTACTCGCGAGCTTTCCT	CGGCTGACGTTTGACAGAAG
Fancm	CGCATGCGCTTCTTCTGA	TGCGGCGGGAAGATGAT
Fancn/Palb2	ACGAAGCATCTCTAAGTCATCCAA	TCCCGGCTGCACTGGTT
Fanco/Rad51c	CAGGCCTGCCGACATGA	ACTCCGGAGCTCCCTCAGA
Fancr/Rad51	GTGATGACGTTGCAGCGATAG	GGCGCGCGACTTTCG
Fancs/Brca1	TGGATCTTGTGTTCCGAAAGG	TCTCCGTCGCCAAGGAAAC
Fanct/Ube2t	CCCAGCTCACAGTCGATTTACA	CGCGTATCCGGAATTCTCA
Rtel1<br>(see details in Supp. Fig. 2)	Primer a : AGCATTTAGGCGTGGCTACAC<br>Primer c : ACCTGGCCCAACAGTTATGG	Primer b : CCGCAGTCCGCTGATTG<br>Primer d : GCATCACAAGCCCAACACAA
**Human Gene**	**Forward Primer (5'-3')**	**Reverse Primer (5'-3')**
FANCD2	TGCCCGGCTAGCACAGA	CCCAGCTGAAGGCAATAAGC
FANCI	GGGCGGATCTTGTTGTTACG	TCCAAAAAGCCCGCTCAA
FANCR/RAD51	CCCCGGCATAAAGTTTGAATT	CTTGATCCTGCGCGAGTTTAC
NUSAP1	CCGTCACCTTTTGCATTCCT	GGCTTGGACAGCTGGTGAA

Supplementary Table 3. DNA sequences for primers used in ChIP experiments.
The primer set (c,d) for *Rtel1* and the primers for *NUSAP1* were used for normalizations.

Supplementary References

1. Vassilev, L.T. et al. In vivo activation of the p53 pathway by small-molecule antagonists of MDM2. *Science* **303**, 844-848 (2004).
2. Simeonova, I. et al. Mutant Mice Lacking the p53 C-Terminal Domain Model Telomere Syndromes. *Cell Rep* **3**, 2046-2058 (2013).
3. Lee, B.K., Bhinge, A.A. & Iyer, V.R. Wide-ranging functions of E2F4 in transcriptional activation and repression revealed by genome-wide analysis. *Nucleic Acids Res* **39**, 3558-3573 (2011).
4. Younger, S.T., Kenzelmann-Broz, D., Jung, H., Attardi, L.D. & Rinn, J.L. Integrative genomic analysis reveals widespread enhancer regulation by p53 in response to DNA damage. *Nucleic Acids Res* **43**, 4447-4462 (2015).
5. Muntean, A.G. et al. The PAF complex synergizes with MLL fusion proteins at HOX loci to promote leukemogenesis. *Cancer Cell* **17**, 609-621 (2010).
6. Montes de Oca Luna, R., Wagner, D.S. & Lozano, G. Rescue of early embryonic lethality in mdm2-deficient mice by deletion of p53. *Nature* **378**, 203-206. (1995).
7. Bardot, B. et al. Mice engineered for an obligatory Mdm4 exon skipping express higher levels of the Mdm4-S isoform but exhibit increased p53 activity. *Oncogene* **34**, 2943-2948 (2015).
8. Sengupta, S. et al. Tumor suppressor p53 represses transcription of RECQ4 helicase. *Oncogene* **24**, 1738-1748 (2005).
9. Woo, H.G., Park, E.S., Thorgeirsson, S.S. & Kim, Y.J. Exploring genomic profiles of hepatocellular carcinoma. *Mol Carcinog* **50**, 235-43 (2011).
10. Wurmbach, E. et al. Genome-wide molecular profiles of HCV-induced dysplasia and hepatocellular carcinoma. *Hepatology* **45**, 938-947 (2007).
11. Ragazzon, B. et al. Transcriptome analysis reveals that p53 and {beta}-catenin alterations occur in a group of aggressive adrenocortical cancers. *Cancer Res* **70**, 8276-81 (2010).
12. Giordano, T.J. et al. Molecular classification and prognostication of adrenocortical tumors by transcriptome profiling. *Clin Cancer Res* **15**, 668-676 (2009).
